# HyperXpress: Rapid Single Vessel DNA Assembly and Protein Production in Microliterscale

**DOI:** 10.3389/fbioe.2022.832176

**Published:** 2022-04-01

**Authors:** Darius Leon Zibulski, Niels Schlichting, Johannes Kabisch

**Affiliations:** ^1^ Computer-aided Synthetic Biology, Darmstadt, Germany; ^2^ Department of Biotechnology and Food Science, NTNU, Trondheim, Norway

**Keywords:** rapid prototyping, ligase cycling reaction, rolling circle amplification, cell-free protein synthesis, semi-rational protein engineering

## Abstract

Rapid prototyping of biological functions has the common aim of generating, screening, and selecting variant libraries as quickly as possible. This approach is now to be extended by the HyperXpress workflow, which connects ligase cycling reaction for DNA assembly, multiply-primed rolling circle amplification for signal amplification, and cell-free protein synthesis to a single vessel reaction in the lower µl scale. After substantial optimization of the method a proof-of-principle demonstrating the high flexibility of HyperXpress for semi-rational protein engineering by expanding, reducing, and replacing *β*-strands of three different green fluorescent proteins is described. These single-day experiments resulted in six functional, new-to-nature GFP prototypes.

## 1 Introduction

Prototyping in bioengineering usually starts at the level of digital sequence information, which can be recombined to obtain novel biological functions of genetic systems (Duportet et al., 2014), proteins (Dopp et al., 2019a), or cells (Robinson et al., 2020). Proteins have a special place among them, as they are being used extensively in many areas of industry, chemistry, medicine, and agriculture, which necessitate the rapid and efficient identification of variants with new and improved properties (Cole and Gaucher, 2011). For this protein engineering process, numerous mutagenesis and recombination methods in combination with a wide variety of selection and screening processes are available (Nannemann et al., 2011), whose common endeavor is to select or screen a highly diverse, functional variant library as effectively as possible (Cole and Gaucher, 2011).

In order to reduce the necessary screening effort and still obtain the most functional and diverse library possible, semi-rational, and focused libraries offer an optimal basis. Various methods, such as whole-gene synthesis, site-saturation mutagenesis, and site-directed mutagenesis, are used to generate these libraries (Lutz, 2010). Of these, site-saturation mutagenesis is one of the most prominent methods, as it allows the targeted introduction of mutations through the use of degenerated primers in a PCR. However, it harbors the risk that if the randomized sequence is expanded too much, secondary and tertiary structural elements can be impaired, thus limiting the functionality of the library (Marcheschi et al., 2013). Apart from this, the associated screening campaigns for such focused libraries often include the use of cell lysates (Chen et al., 2010) or cellular protein synthesis and purification (Heinzelman et al., 2009) of the individual variants, which is quite time-consuming and labor-intensive (Chen et al., 2010). Established systems consisting of RCA and CFPS can be used to circumvent these problems in order to amplify the gene library by RCA and express it via CFPS. But the potential of these RCA-CFPS systems has not yet been fully exhausted, as their practical implementation is still accompanied by cumbersome work steps and increased material consumption (Dopp et al., 2019b). Thus previous RCA-CFPS systems suffer from high total reaction volumes of 15 μl, long execution times of at least 12 h, a high number of workflow steps of six or more and changing of reaction vessels (Dopp et al., 2019b; Hadi et al., 2020).

**FIGURE 1:**
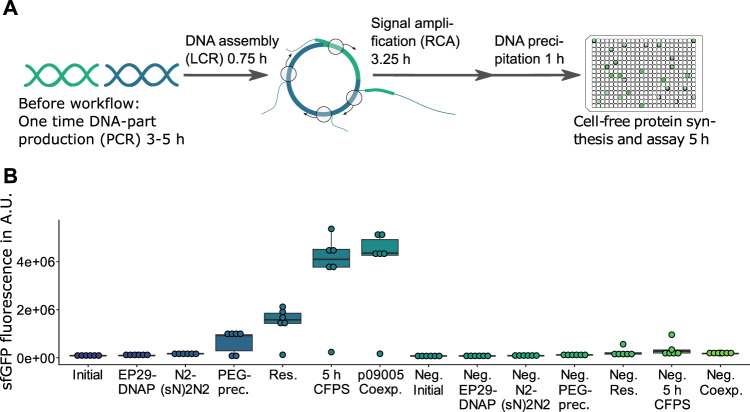
HyperXpress workflow and effect on product formation of the most relevant optimizations. **(A)** The HyperXpress workflow starts with the assembly of DNA parts using LCR. This assembly includes a circularization of the DNA construct which upon addition of the components required for RCA results in an amplification of circular constructs. In order to provide optimal reaction conditions for CFPS a buffer exchange is achieved through DNA precipitation and resuspension. Upon addition of the components required for CFPS each DNA assembly is assayed for product formation. **(B)** Results of the sequential optimizations of the workflow compared to the corresponding 0 nM bridging oligo reaction as a negative control (Neg.). EP29: EquiPhi29™ DNA Polymerase; N2(sN)2N2: endonuclease protected random hexamer oligonucleotides; PEG prec.: in-well DNA precipitation using polyethylenglycol; res.: resuspension of the DNA precipitate in ultrapure water; p09005 coexp.: bicistronic operon plasmid with sfGFP and mKate2 genes used instead of p10024 template with monocistronic GFP reporter construct (*n* = 6).

As a novel rapid approach to prototyping, the HyperXpress workflow (see Figure 1) represents a partially automated, successive single vessel reaction consisting of ligase cycling reaction (LCR) (Kok et al., 2014; Schlichting et al., 2019), multiply-primed rolling circle amplification (RCA) (Dean et al., 2001), and cell-free protein synthesis (CFPS) (Sun et al., 2013; Kwon and Jewett, 2015) within one 384-well plate on the lower microliter scale (Zibulski, 2019). At the beginning of the workflow, the LCR mediates a non-homologous assembly of selected DNA fragments to form a circular product (Kok et al., 2014), which is amplified in the subsequent RCA to form complex dsDNA concatamers (Dean et al., 2001), serving in the final CFPS as a template for the gene expression of the encoded proteins (Chong, 2014). After substantial optimization of this workflow in respect to achieving a low-volume, single vessel reaction of the RCA with LCR and CFPS, semi-rational protein engineering was carried out by LCR-based non-homologous recombination within the GFP protein family in order to work out the advantages and disadvantages of HyperXpress. As a result, deletion, substitution, and expansion of the GFP-*β*-barrel by *β*-strands between the GFP wild type superfolder GFP [sfGFP (Pédelacq et al., 2006)], mAvicFP1 [AF (Lambert et al., 2020)], and mNeonGreen [NG (Shaner et al., 2013)] could be demonstrated in a semi-rational fashion, resulting in 51 new-to-nature sequence variants, six of which were functional.

## 2 Results and Discussion

### 2.1 Development of the HyperXpress Workflow

A traditional workflow for testing new biological functions includes assembling DNA and its transformation into a heterologous host, both for the purpose of selecting and amplifying a circularized DNA assembly as well as producing and analyzing the resulting product. This evaluation of assembled genetic information can be rapidly sped up by using cell-free gene-expression systems (Silverman et al., 2020). HyperXpress further speeds up the path from DNA part to prototype by allowing the combining of all steps in lower µl volume, single vessel reactions. LCR (Kok et al., 2014) is used for DNA assemblies as it allows scarless recombination of DNA parts, while RCA is used to selectively amplify successful and thus circularized assemblies, a function usually performed by a host organism. Supplementary Figure S1 demonstrates the required RCA-amplification of circular constructs. Supplementary Figure S2 with 1:1 vector-to-insert ratios demonstrates that the addition of bridging oligos resulting in circularizing provides a signal an order of magnitude higher than the corresponding non-assembled, linear fragments. This figure as well stresses the importance of later discussed control criteria: increasing ratios of functional expression fragments results in fluorescence signals comparable to the negative controls (ratios of 1:3 and 1:4).

Large batch-to-batch variations of CFPS with respect to its productivity (Supplementary Figure S3) can occur (Takahashi et al., 2015). In order to obtain batch-consistent data by using only one batch for a large prototyping campaign as well as to reduce material consumption, a protocol was developed that allows a 3.6 µl CFPS. Compared to the RCA-CFPS system with the previous CFPS volume minimum of 15 µl (Dopp et al., 2019b), this protocol reduces the required CFPS mix 4.2 fold with a lower necessary working concentration and increases the number of reactions available per batch to over 4,800 reactions for 4 ml of *E. coli* cell extract. The initially employed GenomiPhi™ V2 DNA Amplification Kit does not allow for an easy adjustment of volumes, as reaction volumes are pre-defined and while resulting in strong signals this solution is costly. The kit was thus replaced with a less costly, self-made RCA solution. As shown in Supplementary Figure S4, the phi29-based RCA mix described in this work provided a sufficient signal-to-background ratio and reduced the price by roughly 50%. Improvements to this custom kit came from using phi29 DNA polymerase with its supplied buffer in combination with an expanded RCA incubation time of 180 min as well as the utilization of a pyrophosphatase (PPase) for the removal of RCA-inhibiting pyrophosphate from the reaction (Dean et al., 2001).

### 2.2 Optimization of the HyperXpress Workflow

Next, the sfGFP-gene of the reporter-operon was split into seven parts and a workflow with a sufficient signal-to-background ratio for detecting low activity GFP variants was built up. Moreover, a genetic system producing both an internal reference mKate2 parallel to GFP and a higher sensitivity for GFP variants with low activity was realized. The relative fluorescence used to evaluate the experiments is defined as the $\frac{\text{mean fluorescence of the sample}}{\text{mean fluorescence of the corresponding negative control}}$ (Duportet et al., 2014). All optimizations were performed sequentially and not as a full factorial, so no conclusion can be made about potential combinatorial effects. The results of the optimizations of the workflow are summarized in Figure 1 as well as additional figures in the supplement (Supplementary Figures S5−S8, S10, S12).

The rolling circle amplification step of the workflow was examined with respect to the optimal temperature as well as the utilization of random hexamers required for priming the polymerization reaction. Moreover, two phi-polymerases were tested to achieve a higher RCA efficiency: The wild type polymerase with a temperature optimum at 30°C (phi29 DNA Polymerase, NEB) and an engineered variant with an optimum at 40°C (EquiPhi29™, ThermoScientific). No significant difference in performance could be observed (Supplementary Figure S5, t test: *p* = 0.158 > 0.05). Future experiments will be performed with EquiPhi29™ because a higher stability and a lower error rate than the wild type DNA polymerase are reported (Povilaitis et al., 2016). For the optimization of the priming of the polymerization reaction, both different lengths and protection strategies for the random oligos added to the RCA reaction were tested. Oligos ranging in length from six to nine nucleotides were examined (Supplementary Figure S6). Since an increase in length did not result in a better signal-to-background ratio, less costly hexamers were chosen for the workflow. Both, the wild type phi29 as well as the EquiPhi29™, exhibit prominent 3′-5′-exonuclease activity as part of the proof-reading activity (Salas et al., 2008; Stockbridge and Wolfenden, 2011; Povilaitis et al., 2016), thus protecting the priming hexamers by incorporating 5′-phosphorothioate modifications in different positions was tested (Supplementary Figures S7, S8). Random hexamers with modifications of the two central oligonucleotides (N2sN2N2) showed the strongest increase in signal. It is likely that this central position of the phosphorothioate linkages allows, on the one hand, the degradation of non-annealed 3′-nucleotides of partially-annealed hexamers to generate an elongatable dsDNA-3′-terminus and, on the other hand, protection from complete exonucleolysis of non-annealed hexamers.

Variating the amount of RCA-reaction used in the subsequent CFPS indicated that components contained in the RCA reaction have an inhibiting effect on the CFPS (Supplementary Figure S9). Therefore a potentially automatable buffer exchange based on DNA precipitation and re-suspension in 384 microwell plates (MTP) using centrifugation was developed (see detailed protocol in the Supplemental Material). Besides being faster and not requiring cooling of the sample, PEG-precipitation outperformed ethanol precipitation in terms of signal strength when excluding the two failed reactions in the following CFPS (Supplementary Figures S10, S11). Efficient re-suspension was achieved by adding ultrapure water, sealing the MTP with a hydrophobic film and centrifuging the inverted plate at low rpm (Supplementary Figure S12). The precipitation and re-suspension steps frequently result in the failure of one out of every six replicate reactions performed (e.g. Supplementary Figures S10, S12), yet the small scale of the reactions and the high degree of parallelization can compensate for such events by allowing high numbers of replicates. While DNA precipitation strongly increases the signal, it could be omitted by using a CFPS-adapted RCA buffer which substitutes *in vitro* translation-inhibiting chloride anions (Weber et al., 1977) with L-glutamate (Supplementary Figure S13). Finally, extending the reaction time of the CFPS from 2.5 to 5 h doubled the signal strength (Figure 1).

By optimizing the workflow, HyperXpress now offers a number of advantages over existing RCA-CFPS systems. First of all, the sequence of all workflow steps, including DNA assembly, is performed in the same reaction vessel, while existing systems have to change the reaction vessel between each step. In addition, the CFPS volume has been reduced to 3.6 µl, which results in lower material consumption compared to other RCA-CFPS systems with a minimum final CFPS volume of 15 µl. This results in costs below 0.35 € per 3.6 µl reaction (price calculation see Supplementary Data) for the full workflow, compared to 0.20 € per 15 µl which includes only RCA-CFPS and excludes the price for three required DNA purification steps (Dopp et al., 2019a; Dopp et al., 2019b). Besides, HyperXpress only comprises a minimum of four steps, including DNA precipitation, in contrast to other systems requiring six or eight steps, including several necessary purification or dilution steps (see Supplement for comparison). As a consequence, this workflow can be more readily scaled and, by omitting the precipitation step, which reduces the signal-to-background ratio, can be readily automated. Moreover, the LCR as the first step of the workflow directly enables DNA recombination and circularization to create its own focused DNA libraries and RCA templates, whereas other systems depend on pre-synthesized DNA libraries for starting. Finally, starting with a DNA-part library, HyperXpress can be executed in 10 h compared to at least 12 h (Kumar and Chernaya, 2009; Dopp et al., 2019a; Dopp et al., 2019b; Hadi et al., 2020).

**FIGURE 2:**
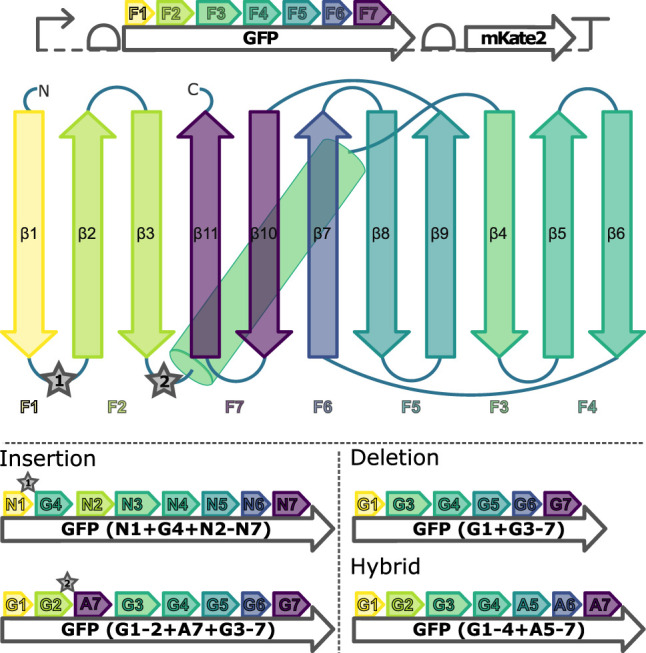
Genetic construct and secondary structure indicating the fragments used for assemblies of the GFP proteins. *Top:* Bicistronic operon structure encoding the engineering target GFP and the assembly-control reporter mKate2. *Middle:* Schematic depicting the F1-F7 GFP DNA fragments used for the family shuffling. mKate2: red fluorescent protein; *β*1-11: *β*-sheets of the GFPs; ★ 1 and 2: insertion sites for additional *β*-sheets. *Bottom:* Examples for the three fragment recombination strategies performed including their nomenclature. G*n*: sfGFP fragment *n*; A*n*: mAvicFP1 fragment *n*; N*n*: mNeonGreen fragment *n*.

To enable discrimination in engineering experiments between reactions without GFP fluorescence due to a failed LCR and reactions without GFP fluorescence due to assembling a genetic construct of non-functional GFP, the operon structure depicted at the top of Figure 2 was designed and demonstrated (Supplementary Figure S14).

Testing the co-expression of sfGFP as the target to be assembled and mKate2 as the LCR-control resulted in a sfGFP signal comparable to that of a monocistronic operon with only a sfGFP gene (Figure 1, p10024 vs. p09005). This sfGFP-mKate2 co-expression without signal loss of sfGFP indicated that the resources in the CFPS (e.g., amino acids, NTPs) and the gene expression rate were sufficient to express mKate2 in parallel to sfGFP.

This operon was used to apply two control criteria to the proof-of-principle described below: At least 2 mKate2 fluorescence units were measured (see Equation 1), and thus the LCR was considered successful, thus the screening result was mKate2^*pos*^. Otherwise, the LCR was deemed to have failed and the screening result was considered mKate2^*neg*^ (Supplementary Figure S14). This is the necessary criterion. A relative sfGFP fluorescence of at least 2 suggests an active GFP variant and thus a GFP^*pos*^ result. In this respect, a relative sfGFP fluorescence of less than 2 causes an inactive variant and thus a GFP^*neg*^ result. This is a sufficient criterion.

### 2.3 Proof-Of-Principle: GFP Domain Shuffling

Based on the optimized workflow, a LCR-based non-homologous recombination between the GFP wild types sfGFP, AF, and NG is carried out, involving the semi-rational insertion, deletion, and substitution of *β*-strands of the GFP-*β*-barrel. For this purpose, all three GFP wild types are divided into 7 fragments according to amino acid sequence homologies and, in the case of sfGFP and NG, additionally according to crystal structure similarities (Pedelacq et al., 2005; Clavel et al., 2016), so that all fragments always carry complete secondary structural elements (Figure 2).

**FIGURE 3:**
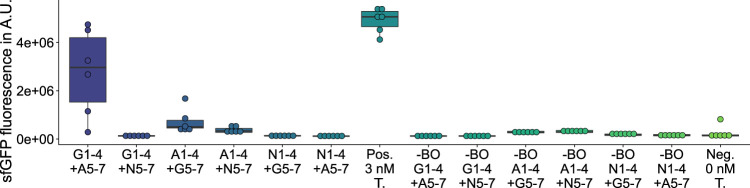
GFP measurements of assemblies with hybrid sequences. G*n*: sfGFP fragment *n*; A*n*: mAvicFP1 fragment *n*; N*n*: mNeonGreen fragment *n*; Pos.: 3 nM of the template plasmid used to obtain the vector for assembly; -BO: no bridging oligos added; Neg.: No Template DNA added.

**FIGURE 4:**
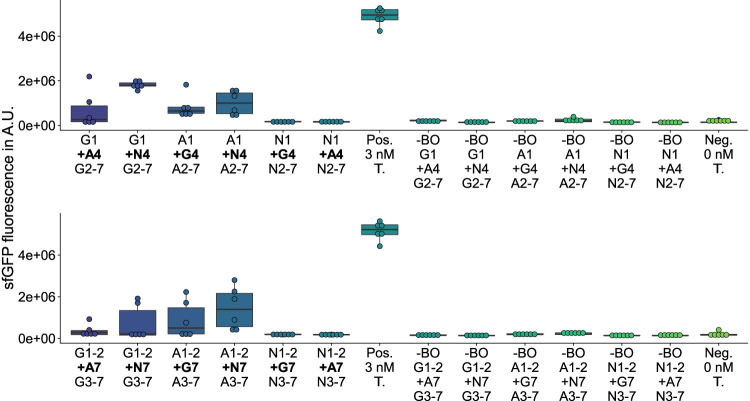
GFP measurements of assemblies with additional sequences inserted. *Top:* Insertion of an additional fragment 4 between fragment 1 and 2. *Bottom:* Insertion of an additional fragment 7 between fragment 2 and 3. G*n*: sfGFP fragment *n*; A*n*: mAvicFP1 fragment *n*; N*n*: mNeonGreen fragment *n*; Pos.: 3 nM of the template plasmid used to obtain the vector for assembly; -BO: no bridging oligos added; Neg.: No Template DNA added.

**FIGURE 5:**
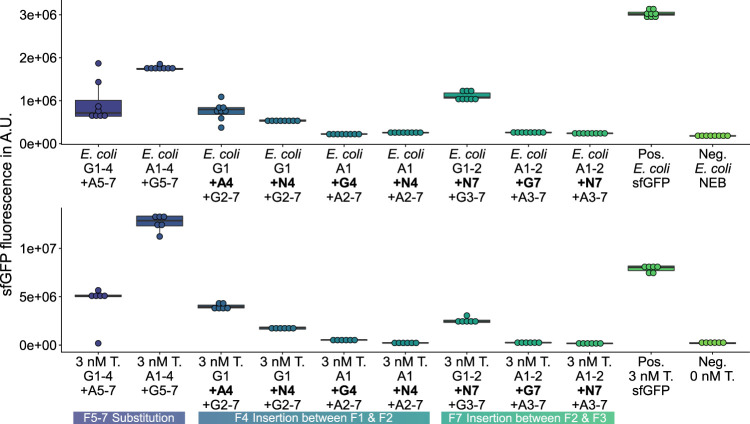
GFP measurements validating constructs previously assembled and screened with HyperXpress. *Top: E. coli in vivo* validation of functional GFP-variants Pos.: *E. coli* expressing template plasmid; Neg.: *E. coli* carrying no plasmid (*n* = 8). *Bottom: in vitro* validation using 3 nM of isolated plasmid DNA (T.) instead of the LCR in the HyperXpress workflow; Neg.: No Template DNA added (*n* = 6).

On the one hand, among the 27 deletion variants, any deletion of fragments 2 (Supplementary Figure S14), 4 (Supplementary Figure S15), or 6 (Supplementary Figure S16) leads to a complete loss of GFP fluorescence activity, which is likely caused by excessive interference (Liu et al., 2015) of the *β*-barrel structure. On the other hand, among the 12 substitution (Figure 3; Supplementary Figures S17, 18) and 12 insertion variants (Figure 4; Supplementary Figures S19, S20), nine new-to-nature GFP variants with GFP fluorescence activity can be identified. Yet four of the nine positive variants need to be categorized as mKate2^*neg*^-GFP^*pos*^ due to their failure to fuly meet the control criteria. Only the *in vivo* and *in vitro* validations (Figure 5; Supplementary Figures S21 and S22) confirm these four mKate2^*neg*^-GFP^*pos*^ variants as active. Besides, among all 51 insertion, deletion, and substitution variants, there are 18 mKate2^*neg*^-GFP^*neg*^ variants as a result of a non-functional LCR, which means that 35.3% of all variants are not even accessible for an evaluation of the fluorescence activity due to insufficient LCR efficiency.

There are also 24 mKate2^*pos*^-GFP^*neg*^ variants, for which no activity can be detected. In spite of this, the workflow produces 9 mKate2^*pos*^- and mKate2^*neg*^-GFP^*pos*^ GFP variants, of which the *in vivo* and *in vitro* validations show four insertion and two substitution variants to be classified as active, while the remaining three insertion variants cannot be definitively evaluated because of the existence of point mutations at the time of the validations. Overall, the HyperXpress workflow yields 11.8% of validated active GFP variants from a focused library of 51 variants.

The three validated, active sfGFP insertion variants suggest that the sfGFP-*β*-barrel allows the homology-independent insertion of the AF-or NG-fragment 4 or 7 (see insertion examples in Figure 2) C-terminal to the sfGFP-fragment 1or 2 without complete loss of activity. At the same time, there is an active AF insertion variant with an insertion of the sfGFP-fragment 4 C-terminal to the AF-fragment 1, which suggests a milieu-dependent loss of activity being only active *in vitro* in the context of CFPS of the HyperXpress workflow. Otherwise, there are two active substitution variants where the sfGFP- respectively AF-fragments 5 to 7 are substituted by the corresponding homologous AF-respectively sfGFP-*β*-strands. This manifests itself as an amino acid sequence homology dependency of this kind of substitution.

## 3 Conclusion

HyperXpress enables rapid prototyping as a single vessel *in vitro* workflow by enabling flexible, combinatorial DNA assembly using LCR, selection and amplification of correctly circularized DNA via RCA, and protein production using cell-free protein synthesis. The extensively optimized reaction steps and reagents allow down-scaling to low µL volumes, which allows a batch consistent and affordable throughput of thousands of samples. As a proof-of-concept, the HyperXpress workflow was used for the first time in semi-rational protein engineering and allows a fast, partially automated, material-saving screening of new GFP variants. Future work will include semi-rational protein engineering in other protein families, the engineering of multi-protein complexes, the construction of new metabolic pathways, and the design of genetic circuits (Gregorio et al., 2019).

## 4 Materials and Methods

A detailed step-by-step protocol for the optimized HyperXpress workflow is provided in the Supplementary Material. A table of the utilized oligonucleotides can be found in Supplementary Table S1 (bridging oligos) and Supplementary Table S2 (primers). Strains are listed in Supplementary Table S3. Plasmid p09008 containing the hybrid-GFP (A1-4+G5-7), which showed a very high *in vitro* signal and can also be used to combine arbitrary LCR assemblies with mKate2 as a control, is deposited at Addgene (#173715).

### 4.1 5′-Phosphorylation and PCR Amplification

The 5′-phosphorylation of the DNA primers [modified after Schlichting et al. (2019), ordered lyophilized and desalted; Sigma-Aldrich] takes place in a 50 µl reaction mixture consisting of 10 µM DNA primers, 1x T4-PNK/T4-polynucleotide kinase buffer, 0.2 U/µl T4-PNK (New England BioLabs) and 1.7 mM ATP. This phosphorylation mixture is incubated for 70 min at 37°C, followed by inactivation for 20 min at 65°C. The 5’-phosphorylated primers are stored at −20°C and can be used in PCRs without further purification. Afterwards, all DNA fragments are amplified via 50 µl PCRs consisting of 0.2 mM of each dNTP, 1x Q5® Reaction Buffer Pack, 0.04 U/µl Q5® High-Fidelity DNA Polymerase (New England BioLabs), 250 nM of each 5’-phosphorylated forward and reverse primer, and 1 fM of template DNA (0.01 fM for the vector amplification). After the PCR, the PCR mixtures are digested with DpnI (1x CutSmart® Buffer, 0.4 U/µl DpnI; New England BioLabs) for 1 h at 37°C and denatured for 20 min at 80°C. Finally, the DpnI digested mixtures are purified via the innuPREP PCRpure Kit (Analytik Jena GmbH) and analyzed photometrically with regard to DNA concentration and quality (UV5 Nano; Mettler Toledo). The purified DNA fragments are stored at −20°C.

### 4.2 Ligase Cycling Reaction

In each well of a 384-well plate, 0.612 µl of LCR mixture is placed, which consists of 3 nM per DNA fragment, 30 nM per BO (ordered lyophilized and desalted; Sigma-Aldrich), 1x Ampligase® buffer [self-made, see Schlichting et al. (2019) or protocol in the Supplementary Material], 10 mM MgCl2, 0.5 mM NAD^+^, and 0.3 U/µl Ampligase® (Lucigen) [modified after Schlichting et al. (2019)]. This LCR mixture is cycled through the following temperature program: 1. 92°C for 2 min; 2. 92°C for 5 s; 3. 66°C for 90 s; 4. repeat steps 2 and 3 another 24 times.

### 4.3 Rolling Circle Amplification

The multiply-primed RCA (modified after Dean et al., 2001) is divided into three sub-steps: annealing, isothermal amplification, and inactivation. For the annealing, a 0.468 µl solution of random N2(sN)2N2 DNA primers (ordered lyophilized and desalted; Sigma-Aldrich) and 10x phi29 DNA Polymerase Reaction Buffer/phi29 buffer (New England BioLabs) is dispensed to the 0.612 µl LCR via a contactless nanoliter-dispenser (I.DOT, DISPENDIX) so that the resulting 1.08 µl annealing mixture consists of 56.7% (v/v) LCR, 166.7 µM random hexamers, and 1.67x phi29 buffer. That annealing mixture is denatured for 3 min at 95°C and then cooled down to 4°C as quickly as possible. This is followed by the isothermal amplification, for which a 0.72 µl solution of dNTPs, BSA (New England BioLabs), EquiPhi29™ DNA polymerase (Thermo Scientific), inorganic *E. coli* pyrophosphatase (New England BioLabs), and DTT is dispensed to the 1.08 µl annealing mixture via the I. DOT. All of that results in the final 1.8 µl RCA mixture consisting of 34% (v/v) LCR, 100 µM N2(sN)2N2, 1 mM of each dNTP, 0.4 μg/μl BSA, 1x phi29 buffer, 0.36 U/µl EquiPhi29™ DNA polymerase, 0.004 U/µl inorganic *E. coli* pyrophosphatase, and 4 mM DTT. This 1.8 µl of RCA mixture is incubated for 3 h at 40°C, followed by inactivation for 10 min at 65°C.

### 4.4 384-Well DNA Precipitation and Re-Suspension

At the beginning of the PEG DNA precipitation [modified after Paithankar and Prasad (1991)] carried out at room temperature, a 1.8 µl solution of 26% (w/v) PEG-8000 and 20 mM MgCl2 is added via a dispenser pipette to the 1.8 µl RCA, resulting in a 3.6 µl precipitation mixture with 50% (v/v) RCA, 13% (w/v) PEG-8000 and 10 mM MgCl2. This mixture is centrifuged at 4,000 rpm at 20°C for 30 min. In order to be able to remove the supernatant from the DNA precipitate, the 384-well plate is covered with a piece of paper and briefly centrifuged in an inverted position in the table centrifuge for a few seconds so that the supernatant is centrifuged out of the wells onto the paper. Afterwards, a washing step is executed in which 3.6 µl of 70% (v/v) ethanol is dispensed into the wells and centrifuged for 5 min at 4,000 rpm and 20°C, followed by discarding the supernatant by centrifugation in the inverted position on the table centrifuge. That washing step is repeated once. The washed DNA precipitate is then dried for 10 min at 30°C; 1.8 µl ultrapure water is added via the nanoliter-dispenser and dissolved again at 50°C for 10 min. To resuspend the DNA solution, the 384-well plate is tightly closed with Parafilm (Pechiney Plastic Packaging). This sealed 384-well plate is centrifuged in an inverted position for a few seconds in a table-top centrifuge, resulting in the collection of the solution on the Parafilm lid of the wells. The plate is then centrifuged again in the non-inverted position for a few seconds to transfer the solution back into the wells.

### 4.5 Cell-Free Protein Synthesis

To carry out the 3.6 µl CFPS [modified after Sun et al. (2013)], a 1.8 µl solution of *E. coli* cell extract (CE, self-made, see protocol in the Supplementary Material) and cell extract buffer (CEB) is dispensed via the nanoliter-dispenser to the 1.8 µl DNA solution to get 50% (v/v) DNA solution, 22.73% (v/v) CE and 27.27% (v/v) CEB (under permanent cooling of the 384-well plate). In contrast to Sun et al. (2013), the CE is produced via modified cell cultivation and the cell lysis is done via sonification (see protocol in the Supplementary Material). This CFPS mixture is covered with an optical adhesive cover, incubated for 5 h at 29°C in a microplate reader (ClarioStar Plus, BMG LabTech) and the fluorescence is measured online via top optic with the following wavelength settings: sfGFP and AF (*λ*ex = 470 ± 7.5 nm/*λ*em = 515 ± 10 nm; mKate2 (*λ*ex = 588 ± 7.5 nm/*λ*em = 633 ± 10 nm); NG (*λ*ex = 491 ± 7 nm/*λ*em = 533 ± 10 nm).

### 4.6 Sequence Verification

All DNA assemblies described have been verified by sequencing. For this, 50 µl electro-competent *E. coli* (*E. coli* NEB®-10*β*, New England Biolabs) were mixed with 5 µl LCR mixture, filled into an electroporation cuvette and incubated for 5 min on ice. These cells were electroporated for 4.6 ms at 2.5 kV, resuspended in 1 ml of LB media and incubated for 1 h at 37°C and 200 rpm. Finally, the cells were plated out on amp-containing LB agar plates so that they could grow over night at 37°C. Afterwards, selected colonies were cultivated in amp-containing LB media to create -80°C cryo-stocks for the *in vivo* validation and to obtain plasmids for sequencing and *in vitro* validation.

### 4.7 *In vivo* Validation

An aliquot of the *E. coli* −80°C cryo-stock is plated out on ampicillin (amp)-containing LB agar plates and incubated at 37°C overnight. The next day, the grown *E. coli* colonies are used to inoculate an overnight ampicillin culture, which is incubated at 37°C and 200 rpm. From this overnight culture, an OD_*600*_ of 0.5 is set in 1.5 ml of LB-amp medium (preheated to 30°C), representing the final culture to be measured. The final culture is distributed in 8-fold repetitions with 150 µl per well over a 96-well plate. These 150 µl final cultures are incubated for 5 h at 30°C and 200 rpm using 3D-printed plate holders (Bruder et al., 2019) so that after 5 h an end point measurement can be carried out in a microplate reader for the absorption at 600 nm and for the sfGFP, mKate2, and NG fluorescence. Before the first measurement, the plate is shaken for 15 s at 200 rpm.

### 4.8 Software

Plots were generated using R (R Development Core Team, 2008) and R-Studio (RStudio Team, 2018) as an IDE. *In silico* planning was done using Geneious R10.2 (https://www.geneious.com). Combinatorial DNA assemblies were planed using DIVA (https://public-diva.jbei.org/) with custom add-ons for automatic design of bridging oligos as well as automated generation of dispensing protocols (yet unpublished work).

## Data Availability

The original contributions presented in the study are included in the article/Supplementary Material, further inquiries can be directed to the corresponding author.
