# Supplementary material for: HyperXpress: Rapid Single Vessel DNA Assembly and Protein Production in Microliterscale
Source: Front Bioeng Biotechnol. 2022 Apr 1;10:832176. doi: 10.3389/fbioe.2022.832176 (PMC9011061; doi:10.3389/fbioe.2022.832176)
Supplement: Supplementary file 1 [file DataSheet2.pdf]

Protocol for execution of the HyperXpress workflow

The protocol for the **HyperXpress workflow** comprises four steps **carried out in the same well** of a 384-well-plate:

1. LCR / ligase cycling reaction

2. Multiply-primed RCA / rolling circle amplification

3. PEG / polyethylene glycol DNA precipitation

4. CFPS / cell-free protein synthesis

Important: Currently the protocol has only been applied using a nanoliter dispenser (i.e. dispendix I.DOT). Other nanoliter dispensers such as Labcyte Echo should work but have not been tested. Scaling to volumes which can be handled by µl-pipettes might be possible but has not been tested.

It is necessary to use a **thermocycler compatible** 384-well-plate with a **V-bottom** and a **maximum volume of 25 µl** per well. Higher maximum volumes per well will increase the risk for evaporation of the reaction mixtures during thermal cycling.

Notes: For thermal cycling of the 384-well-plate, a flat, thermally stable silicone mat should always be placed between the 384-well-plate and the lid of the thermocycler so that the wells are tightly closed on the lid.

Reagents for the preparation and execution of the HyperXpress workflow

Reagents for the production of the *E. coli* cell extract:

- 2xYT+P media (see Sun *et al*. (2013)[1])

- S30A buffer (see Sun *et al*. (2013)[1])

Reagents for the production of the cell extract buffer:

- L-Glutamic acid hemimagnesium salt tetrahydrate (Mg-glutamate)

- L-Glutamic acid potassium salt monohydrate (K-glutamate)

- Amino acid solution (see “II. Production of the cell extract buffer”)

- Energy solution (see Sun *et al*. (2013)[1])

- 1,4-dithiothreitol (DTT)

- PEG-8000

Reagents for the 5’-phosphorylation of PCR primers:

- T4-polynucleotide kinase / T4-PNK (New England BioLabs®)

- 10x T4-PNK buffer (New England BioLabs®)

- ATP

- DNA primer (ordered desalted and dried from Sigma-Aldrich)

Reagents for the PCR amplification of the DNA fragments:

- Proof-reading DNA polymerase that produces blunt ended DNA fragments

- DpnI (New England BioLabs®)

- 10x CutSmart® Buffer (New England BioLabs®)

Reagents for the LCR:

- BO / bridging oligos (ordered desalted and dried from Sigma-Aldrich)

- 10x Ampligase® buffer (self-made)

- $MgCl_2$

- NAD^+

- Ampligase® (Lucigen)

Reagents for the RCA:

- $N_2(sN)_2N_2$ random primer
  (ordered as modified DNA primers in a desalted and dried form from Sigma-Aldrich)

- 10x phi29 DNA Polymerase Reaction Buffer (New England BioLabs®)

- dNTP

- BSA, Molecular Biology Grade (New England BioLabs®)

- EquiPhi29™ DNA polymerase (Thermo Scientific)

- *E. coli* pyrophosphatase, inorganic (New England BioLabs®)

- DTT

Reagents for the PEG DNA precipitation:

- PEG-8000

- $MgCl_2$

- Ethanol (≥99.8%)

Reagents for the *E. coli* cell extract-based CFPS:

- *E. coli* cell extract (see “I. Production of E. coli cell extract”)

- Cell extract buffer (see “II. Production of the cell extract buffer”)

0. Preparations for the HyperXpress workflow

Before the HyperXpress workflow can be executed, some preparations have to be done:

- Production of *E. coli* cell extract

- Production of the cell extract buffer

- 5'-Phosphorylation of PCR primers

- PCR amplification of the DNA fragments

I. Production of *E. coli* cell extract

The **production of the *E. coli* cell extract** (modified after Sun *et al*. (2013)[1]) is carried out as follows:

1. Cell cultivation

1.1. Plating out *E. coli Rosetta EL22*

1. Plating out an aliquot of the *E. coli Rosetta EL22* (-80°C) cryo-stock on an LB agar plate containing chloramphenicol / Chl (final: 34 µg/ml) and kanamycin / Kan (final: 50 µg/ml)

   Notes: The commercial available *E. coli* strain *E. coli BL21-Rosetta2* can be used instead of the laboratory internal strain *E. coli Rosetta EL22*.

2. Incubate the plated cells at 37°C until colonies are visible (8-12 h)

   Notes: It is possible to carry out the steps "1.1. Plating out *E. coli Rosetta EL22*" and "1.2. Creating the overnight culture / preculture" on the same day, if step 1.1 is executed in the morning and step 1.2 in the evening.

1.2. Creating the overnight culture / preculture

1. Mix 50 ml 2xYT+P medium with 50 µl of 34 mg/ml Chl and 50 µl of 50 mg/ml Kan in a 250 ml flask (final: 34 µg/ml Chl and 50 µg/ml Kan)
2. Inoculate the solution with a colony of *E. coli Rosetta EL22*
3. Incubate the inoculated solution at 200 rpm and 37°C overnight
   (ideal: 16-18 hours)

1.3. Creating the final culture

1. Prewarm the 2xYT+P medium overnight to 37°C (start the day before)
2. Mix 800 ml 2xYT+P medium with 800 µl of 34 mg/ml Chl and 800 µl of 50 mg/ml Kan in a 5 liter flask (final: 34 µg/ml Chl and 50 µg/ml Kan)
3. Inoculate the 800 ml solution with the entire 50 ml overnight culture
4. Incubate the inoculated solution at 200 rpm and 37°C until an OD_{600} of 3-3.5 is reached

   Notes: The incubation must not last longer than 4 hours.

Important: All samples in step **"2. Cell harvest"** should be constantly cooled **on ice**!

2. Cell harvest

1. Distribute the final culture into four 500 ml centrifuge beakers
2. Centrifuge the final culture at 5000 xg for 12 min at 4°C
3. Decant off and discard the supernatant so that a pellet remains

   Note: Drain remaining liquid from the bottles on a sterile cloth
4. Add 1 ml of 1 M DTT (use fresh aliquots) to the 500 ml S30A buffer

   Important: Permanent cooling of the (DTT-added) S30A buffer
5. Add 40 ml of DTT-added S30A buffer to each beaker
6. Shake (or vortex) the beakers until the pellet is completely resuspended
7. Centrifuge the beakers at 5000 xg for 12 min at 4°C
8. Decant off and discard the supernatant so that a pellet remains

   Note: Drain remaining liquid from the bottles on a sterile cloth
9. Repeat steps 5-8, but after resuspending in the repeated step 6, two suspensions each are combined in a single centrifuge beaker
10. Add 20 ml of DTT-added S30A buffer to each beaker
11. Shake (or vortex) the beakers until the pellet is completely resuspended
12. Weigh two sterile 50 ml plastic tubes and label them accordingly
13. Transfer the two pellet buffer suspensions into the two weighed 50 ml tubes
14. Centrifuge the plastic tubes at 3500 xg for 20 min at 4°C
15. Decant off and discard the supernatant
16. Centrifuge the plastic tubes at 2000 xg for 2 min at 4°C

17. Pipette off and discard any remaining liquid
18. Weigh the two pellet-filled plastic tubes
19. Add the calculated volume of DTT-added S30A buffer to each pellet

    Formula: $\mathrm{V(S30A)} = \mathrm{m(pellet)} \cdot \frac{\mathrm{ml}}{\mathrm{g}}$; m(pellet) in g ; V(S30A) in ml
20. Vortex the two pellet buffer suspensions until they are homogeneous and afterwards centrifuge very briefly (approx. 60 xg for 30 s at 4°C) to collect the suspension at the bottom
21. Transfer all pellet buffer suspensions by pipette into a single 50 ml plastic tube and afterwards distribute them onto six 2 ml microcentrifuge tubes (approx. 1.1 ml per tube)

    Important: Cut off the front pipette tip to reduce shear forces

3. Cell lysis

All work in **“3. Cell lysis”** only takes place **on ice water**!

1. Create an ice water bath for the suspension-filled 2 ml tubes
2. Sonify the six suspensions

   Sonification parameters: 10 s pulse-on, 10 s pulse-off, power of 4-5 W per pulse, energy up to 600 J, amplitude of 50%, 6 min per tube

   Important: The **2 ml tubes** have to be **immersed almost completely in the ice water** so that the suspension does not overheat.

   Notes: The sonification probe must be positioned in the center of the suspension. Make sure your sonification probe is not used up / porous.

   After successful sonification, the suspension appears clear yet viscous.

4. Isolation of the cell extract

All work **up to and including step 3** takes place only **on ice**!

1. Centrifuge the sonicated suspensions at 12000 xg for 10 min at 4°C
   => Separation of cell mass and cell extract
2. Transfer the six supernatants with a pipette into four 2 ml microcentrifuge tubes and discard the pellets
   Important: Do not carry over any pellet!
   => Isolation of the **crude cell extract**
3. If the pellet is carried over, the following two sub-steps are executed:
   3.1. Centrifuge the supernatant at 12000 xg for 10 min at 4°C
   3.2. Transfer the new supernatant by pipette into four new 2 ml tubes
4. Incubate the four aliquots at 220 rpm and 37°C for 80 min
   Notes: This incubation at 37°C serves as run-off reaction to release the ribosomes from the endogenous mRNA.

All future steps only take place **on ice**!

5. Centrifuge the solutions at 12000 xg for 10 min at 4°C
6. Collate all aliquots by pipetting all supernatants into a single tube and discard the pellet
   => Isolation of the **final cell extract**
7. Aliquot the solution in PCR tubes (50 µl aliquot per PCR tube)
8. **Shock freeze** the PCR tubes **in liquid nitrogen**
9. Store the PCR tubes at -80°C

The composition and manufacturing process of the 2xYT+P media and the S30A buffer can be found in the original publication of Sun *et al*. (2013)[1].

II. Production of the cell extract buffer

The **cell extract buffer** (based on Sun *et al*. (2013)[1]) has the following composition (see Tab. 1):

Tab. 1: Composition of the cell extract buffer

*Mg-glutamate: L-Glutamic acid hemimagnesium salt tetrahydrate; K-glutamate: L-Glutamic acid potassium salt monohydrate; DTT: 1,4-dithiothreitol

Component	Volume in µl	Final concentration in 1979.26 µl
500 mM Mg-glutamate*	94.26	23.81 mM
3000 mM K-glutamate*	251.37	381 mM
5.33x Amino acid solution	883.72	2.38x
14x Energy solution	336.66	2.38x
100 mM DTT*	70.70	3.57 mM
40% (w/v) PEG-8000	235.66	4.76% (w/v)
MilliQ H_2O	106.89	/
**Sum**:	**1979.26**	

Important: The **5.33x amino acid solution** has the following composition: 1.5 ml each of 168 mM Ala, Arg, Asn, Asp, Gln, Glu, Gly, His, Ile, Lys, Met, Phe, Pro, Ser, Thr, Val, Trp, Tyr, Cys; 1.5 ml of 140 mM Leu; 1.5 ml MilliQ H_2O (final: 8 mM per amino acid except for Leu with 6.667 mM).

The composition and manufacturing process of the 14x energy solution can be found in the original publication of Sun *et al*. (2013)[1]. For the amino acid solution, the composition is changed compared to the original publication of Sun *et al*. (2013)[1] that is listed above under the point "important". But the manufacturing process of the amino acid solution is identical with the original publication[1].

III. 5’-Phosphorylation of PCR primers

The 5’-phosphorylation of the PCR primers is necessary to produce 5’-phosphorylated DNA fragments that can be ligated in the course of the HyperXpress workflow.

The **5’-phosphorylation of PCR primers** (modified after Schlichting *et al*. (2019)[2]) is carried out as follows

1. Create a **phosphorylation mixture** with the following composition (see Tab. 2):

Tab. 2: Composition of a 50 µl phosphorylation mixture

Component	Volume in µl	Final concentration
10 U/µl T4-polynucleotide kinase / T4-PNK	1	0.2 U/µl
10x T4-PNK buffer	5	1x
10 mM ATP	8.5	1.7 mM
100 µM primer	5	10 µM
MilliQ H_2O	30.5	/
**Sum**:	**50**	

2. The phosphorylation mixtures pass through the following **phosphorylation temperature program** (see Tab. 3):

Tab. 3: Phosphorylation temperature program

Step	Conditions
1. Phosphorylation	37°C for 70 min
2. Inactivation	65°C for 20 min
3. Hold	10°C forever

3. Store the phosphorylated primers at -20°C

IV. PCR amplification of the DNA fragments

For the PCR amplification of the DNA fragments, some aspects have to be considered:

- Use a **proof-reading DNA polymerase** that produces **blunt ended DNA fragments** (e.g. Q5® High-Fidelity DNA Polymerase)
- Adjust **250 nM of 5'-phosphorylated primers** (10 µM in the stock solution) in the PCR mixture so that the primer stock solution can be used without a previous purification (e.g. dialysis)
- Utilize a maximum of 1 fM for the amplification of the inserts (combined with 35 PCR cycles) and a **maximum of 0.01 fM** for the amplification of the vector (combined with 40 PCR cycles) to minimize the risk of plasmid carryover

  Important: It is really important to use a minimum of template DNA in the PCR because the DpnI digestion often is insufficient to remove all template.
- After the PCR: **DpnI digestion** of the PCR mixture to degrade the template DNA

  Notes: DpnI digestion is carried out by setting 1x CutSmart® Buffer and 0.4 U/µl DpnI in the PCR mixture and incubating for 1 h at 37°C followed by denaturation for 20 min at 80°C.
- After the PCR and DpnI digestion: Purify the DNA fragments

  Notes: For the purification of the DNA fragments, two PCR mixtures should be purified via one column to increase the concentration of the purified DNA fragments (especially for the vector).

Notes: Avoiding plasmid carryover from PCR is essential, because any circular DNA (even from the PCR) is strongly amplified in the course of the RCA-step of the HyperXpress workflow.

1. LCR / ligase cycling reaction

The **LCR** (modified after Schlichting *et al*. (2019)[2]) is carried out as follows:

1. Every **0.612 µl LCR mixture** has the following composition in Tab. 4 (see Tab. 4)

   Important: The DNA fragments need 5'-phosphorylated ends so that they can be ligated in the LCR.

   Notes: The BOs / bridging oligos are designed via DiVA using the optimal parameters determined by Schlichting *et al*. (2019)[2]. Currently (July 2021) the DiVA version with included LCR is not yet publicly available but should be by the end of 2021. Please check https://public-diva.jbei.org/ for up-to-date versions.

   Notes: Composition of the 10x Ampligase® buffer (self-made): 200 mM Tris-HCl (pH=8.3), 250 mM KCl, 0.1% Triton X-100.

   Alternatively 100 mM $MgCl_2$ can be added to the 10x Ampligase® buffer to dispense with the separate addition of $MgCl_2$. But NAD^+ stock solutions have to be stored separately and aliquoted at -20°C because of its sensitivity to freeze-thaw cycles and light exposure.

   Notes: It is recommended to premix a multiple of 0.0612 µl Ampligase® buffer, 0.0245 µl $MgCl_2$, 0.0306 µl NAD^+, 0.0367 µl Ampligase® and 0.153 µl MilliQ H_2O to a master-mix that is dispensed as 0.306 µl aliquots (50% (v/v) of each 0.612 µl LCR) via a nanoliter dispenser into each well. In the same way, a multiple of the volume of DNA fragments and BOs should be mixed with the remaining volume of MilliQ H_2O in different master-mixes and added to the wells via a nanoliter dispenser or manually via a pipette (DNA and BO master-mixes represent together 50% (v/v) of each 0.612 µl LCR).

   DNA and BO master-mixes should first be filled into the wells and only then the master-mix with all the other components.

Tab. 4: Composition of a 0.612 µl LCR

**Component**	**Volume**	**Final concentration in 0.612 µl**
DNA fragments	x µl	3 nM of each fragment
3 µM of each BO	y µl	30 nM of each BO
10x Ampligase® buffer (self-made)	0.0612 µl	1x
250 mM $MgCl_2$	0.0245 µl	10 mM
10 mM NAD^+	0.0306 µl	0.5 mM
5 U/µl Ampligase®	0.0367 µl	0.3 U/µl
MilliQ H_2O	Fill up to 0.612 µl	/
**Sum**:	**0.612 µl**	

2. Fill the 0.612 µl LCR mixture in the well of a 384-well-plate, centrifuge down on the table centrifuge and **cover it with aluminum cover film**.

   Important: The aluminum cover film prevents the complete evaporation of the LCR mixture during the LCR temperature program.

   Notes: LCRs should not be executed in the columns 1 and 24 or in the rows A and P of the 384-well plate because of a strong edge effect during the LCR temperature cycles that results in the complete evaporation of the LCR mixture. All other rows and columns can be used without any relevant edge effect.

3. All 0.612 µl LCR mixtures pass through the following **LCR temperature program** (see Tab. 5):

Tab. 5: LCR temperature program

**Step**	**Conditions**
1. Initial denaturation	92°C for 2 min
2. Denaturation	92°C for 5 s
3. Annealing and ligation	66°C for 90 s
4. Cycling	Repetition of the steps 2 and 3 another 24 times
5. Hold	10°C forever

Important: This LCR temperature program is not universal for every specific LCR and has to be optimized for complex LCRs (i.e. more than 4 DNA fragments, high GC content, BOs with strong secondary structures). The first variables to optimize are denaturation temperature and time.

2. Multiply-primed RCA / rolling circle amplification

The multiply-primed RCA (modified after Dean *et al*. (2001)[3]) takes place in three steps:

1. Annealing of random primers

2. Isothermal amplification

3. Inactivation of the DNA polymerase

The **annealing of random primers** (first step of RCA) is carried out as follows:

1. Add phosphorothioate protected random hexamer primer / $N_2(sN)_2N_2$ (N: dA, dT, dC or dG; sN: 5'-phosphorothioate protected deoxyribonucleotide) and phi29 reaction buffer to the LCR mixture in the well to form a **1.08 µl annealing mixture** (see Tab. 6)

   Notes: It is recommended to premix $N_2(sN)_2N_2$ primer, 10x phi29 buffer and MilliQ H_2O so that the premix solution can be dispensed to the LCR mixture.

Tab. 6: Composition of a 1.08 µl annealing mixture

**Component**	**Volume**	**Final concentration in 1.08 µl**
LCR mixture	0.612 µl	56.7% (v/v)
1 mM $N_2(sN)_2N_2$	0.18 µl	166.7 µM
10x "phi29 DNA Polymerase Reaction Buffer" / 10x phi29 buffer (from New England BioLabs®)	0.18 µl	1.67x
MilliQ H_2O	0.108 µl	/
**Sum**:	**1.08 µl**	

<u>Notes</u>: The 10x phi29 DNA Polymerase Reaction Buffer from New England BioLabs®, even when using the EquiPhi29™, achieves the best results in the HyperXpress workflow. This buffer has the following 1x composition: 50 mM Tris-HCl, 10 mM $MgCl_2$, 10 mM $(NH_4)_2SO_4$, 4 mM DTT (pH 7.5 at 25°C)[4].

<u>Notes</u>: It is important to use $N_2(sN)_2N_2$ (with the phosphorothioate protection in the middle of the primer hexamers) because the RCA efficiency depends on the position of the phosphorothioate linkage within the primers.

2. Briefly centrifuge down the annealing mixture on the table centrifuge and **cover the 384-well-plate with aluminum cover film**

   <u>Important</u>: The aluminum cover film prevents the complete evaporation of the annealing mixture during the annealing.

3. All 1.08 µl annealing mixtures pass through the following **<u>annealing temperature program</u>** (see Tab. 7):

Tab. 7: Annealing temperature program

**Step**	**Conditions**
1. Denaturation	95°C for 3 min
2. Annealing	4°C forever

<u>Important</u>: From this moment on, the **annealing mixture must be permanently cooled** (on ice) so that the annealing is not reversed.

The **<u>isothermal amplification</u>** (second step of RCA) and the **<u>inactivation of the DNA polymerase</u>** (third step of RCA) is carried out as follows:

1. Add dNTP, bovine serum albumin / BSA, EquiPhi29™ DNA polymerase / EquiPhi29™ DNAP, inorganic *E. coli* pyrophosphatase / *E. coli* iPPase and 1,4-dithiothreitol / DTT to the annealing mixture in the same well (and centrifuged down on the table centrifuge) to create a **<u>1.8 µl amplification mixture</u>**
   (see Tab. 8)

Notes: It is recommended to premix dNTP, BSA, EquiPhi29™ DNAP, *E. coli* iPPase, DTT and MilliQ H_2O so that the premix solution can be dispensed to the annealing mixture.

Notes: DTT has to be added additionally to the amplification mixture because the DTT in the 10x phi29 buffer is completely degraded during the denaturation at 95°C for the primer annealing.

Tab. 8: Composition of a 1.8 µl amplification mixture

**Component**		**Volume**	**Final concentration in 1.8 µl**
Annealing mixture		1.08 µl	60% (v/v)
	LCR mixture		34% (v/v)
	$N_2(sN)_2N_2$		100 µM
	phi29 buffer		1x
10 mM of each dNTP		0.18 µl	1 mM of each dNTP
20 µg/µl BSA		0.036 µl	0.4 µg/µl
10 U/µl EquiPhi29™ DNAP		0.065 µl	0.36 U/µl
0.1 U/µl *E. coli* iPPase		0.072 µl	0.004 U/µl
160 mM DTT		0.045 µl	4 mM
MilliQ H_2O		0.322 µl	/
**Sum**:		**1.8 µl**	

2. All 1.8 µl amplification mixtures pass through the following **amplification temperature program** (see Tab. 9):

Tab. 9: Amplification temperature program

**Step**	**Conditions**
1. Isothermal amplification	40°C for 3 h
2. Inactivation of the DNA polymerase	65°C for 10 min
3. Hold	10°C forever

3. PEG / polyethylene glycol DNA precipitation

Before the precipitation, the **amplification mixture is prewarmed to room temperature** because the whole PEG DNA precipitation is carried out at room temperature.

The **PEG DNA precipitation** (modified after Paithankar *et al.* (1991)[5]) is carried out as follows:

1. Add a premixed PEG-8000 and $MgCl_2$ solution to the amplification mixture in the same well to form a **3.6 µl precipitation mixture** (see Tab. 10)

   Important: The whole **PEG DNA precipitation** takes place **at room temperature**.

   Notes: After adding the premixed PEG-8000 and $MgCl_2$ solution, the precipitation mixture should be centrifuged down on the table centrifuge and covered with a thin foil to prevent that dust or other particles could fall into the mixture.

Tab. 10: Composition of a 3.6 µl precipitation mixture

Component		Volume	Final concentration in 3.6 µl
Amplification mixture		1.8 µl	50% (v/v)
Premixed PEG-8000 and $MgCl_2$ solution		1.8 µl	50% (v/v)
	26% (w/v) PEG-8000		13% (w/v)
	20 mM $MgCl_2$		10 mM
**Sum**:		**3.6 µl**	

2. Centrifuge the precipitation mixture for 30 min at 4000 rpm and 20°C
3. Gently discard the supernatant by inverting the plate on a piece of paper and centrifuging out briefly on the table centrifuge (maximum 240 rpm)

   Notes: The supernatant does not have to be “centrifuged-out” completely. It is more important to keep the DNA precipitate in the well than to discard the supernatant completely.
4. Add 3.6 µl 70% (v/v) ethanol (pre-warmed to room temperature)

5. Centrifuge for 5 min at 4000 rpm and 20°C
6. Gently discard the supernatant by inverting the plate on a piece of paper and centrifuging out briefly on the table centrifuge (maximum 240 rpm)

   Notes: The supernatant does not have to be “centrifuged-out” completely. It is more important to keep the DNA precipitate in the well than to discard the supernatant completely.
7. Repeat steps 4-6
8. Dry the DNA precipitate at 30°C for 10 min
9. Dissolve the precipitate in 1.8 µl MilliQ H_2O at 50°C for 10 min
10. Centrifugation-based resuspending:
    - 10.1. Seal the 384-well plate with Parafilm so that nothing can escape from the wells
    - 10.2. Briefly centrifuge the plate in an inverted orientation so that the DNA solution collects on the Parafilm lid
    - 10.3. Briefly centrifuge the plate in a non-inverted orientation so that the DNA solution collects again in the wells and cool the plate on ice

Notes: In the case of PEG DNA precipitation, it is advisable to use a direct displacement dispenser pipette for adding the highly viscous premixed PEG-8000 and $MgCl_2$ solution.

4. CFPS / cell-free protein synthesis

The ***E. coli* cell extract-based CFPS** (modified after Sun *et al*. (2013)[1]) is carried out as follows:

1. Mix 818 nl *E. coli* cell extract (CE) with 982 nl cell extract buffer (CEB) to produce 1.8 µl of CE-CEB solution while permanently cooling on ice
2. Add 1.8 µl of CE-CEB solution to the 1.8 µl of DNA solution in the well to form a **3.6 µl CFPS mixture** (see Tab. 11) with permanent cooling of the CFPS mixture on ice

Tab. 11: Composition of a 3.6 µl CFPS mixture

**Component**	**Volume**	**Final concentration in 3.6 µl**
DNA solution	1.8 µl	50% (v/v)
CE-CEB solution	1.8 µl	50% (v/v)
E. coli cell extract		22.73% (v/v)
Cell extract buffer		27.27% (v/v)
**Sum**:	**3.6 µl**	

3. Incubate the CFPS mixture for 5 h at 29°C and measure the fluorescence of the mixture through top optic for detecting the synthesis of fluorescent proteins (covered with an optical adhesive cover)

   Important: The incubation time of the CFPS mixture depends on the gene expression rate and the protein that is synthesized. So the incubation time of 5 h is not fixed and can be adopted to the protein of interest.

   Notes: In the case of fluorescent proteins, the incubation time serves both the CFPS and the actual detection of the fluorescent protein. But for non-fluorescent proteins, downstream assays may be necessary.

References

[1]: “Protocols for Implementing an *Escherichia coli* Based TX-TL Cell-Free Expression System for Synthetic Biology” (2013) Zachary Z. Sun, Clarmyra A. Hayes, Jonghyeon Shin, Filippo Caschera, Richard M. Murray, Vincent Noireaux; J Vis Exp. 2013; (79): 50762. DOI: 10.3791/50762

[2]: “Optimization of the experimental parameters of the ligase cycling reaction” (2019) Niels Schlichting, Felix Reinhardt, Sven Jager, Michael Schmidt, Johannes Kabisch;
Synth Biol (Oxf). 2019; 4(1): ysz020. DOI: 10.1093/synbio/ysz020

[3]: “Rapid Amplification of Plasmid and Phage DNA Using Phi29 DNA Polymerase and Multiply-Primed Rolling Circle Amplification” (2001) Frank B. Dean, John R. Nelson, Theresa L. Giesler, Roger S. Lasken;
Genome Res. 2001 Jun; 11(6): 1095–1099. DOI: 10.1101/gr.180501

[4]: Product website of “phi29 DNA Polymerase” by New England BioLabs®
https://international.neb.com/products/m0269-phi29-dna-polymerase#Product%20Information_Properties%20&%20Usage

[5]: “Precipitation of DNA by polyethylene glycol and ethanol.” (1991) K R Paithankar, K S Prasad;
Nucleic Acids Res. 1991 Mar 25; 19(6): 1346. DOI: 10.1093/nar/19.6.1346
