# Supplementary material for: HyperXpress: Rapid Single Vessel DNA Assembly and Protein Production in Microliterscale
Source: Front Bioeng Biotechnol. 2022 Apr 1;10:832176. doi: 10.3389/fbioe.2022.832176 (PMC9011061; doi:10.3389/fbioe.2022.832176)
Supplement: Supplementary file 2 [file DataSheet1.PDF]

HyperXpress - rapid single vessel DNA assembly and protein production in microliterscale: supplemental document

January 3, 2022

Contents

1 Introduction **S1**

2 Figures and Tables **S1**
2.1 Supplemental Figures S1
2.2 Supplemental Table S12

3 Genbank files of Plasmids **S18**
3.0.1 p10024 . . . . S18
3.0.2 p09002 . . . . S21
3.0.3 p09004 . . . . S23
3.0.4 p09005 . . . . S26

4 Comparisons and Costs **S29**
4.1 Method Comparison S29
4.2 Costs Calculations S32

1 Introduction

This supplemental document contains figures referenced in the main manuscript as well as additional material and methods.

2 Figures and Tables

2.1 Supplemental Figures

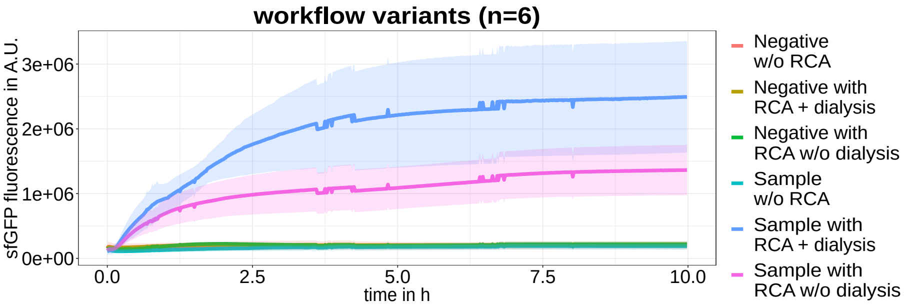

Fig. S 1: Comparison of CFPS for a GFP expression construct with and without prior amplification using RCA. Furthermore dialysis of RCA amplified DNA was examined. Dialysis was carried out by transferring RCA amplified DNA to a micro dialysis membrane (mixed cellulose esters, hydrophilic, 0.025 µm, Merck Millipore) in MilliQ water and incubating for 30 min at room temperature. **Note:** The dialysis of the RCA-amplified DNA for removing CFPS-inhibiting salts was not integrated into the HyperXpress workflow because it would interrupt the desired single vessel reaction

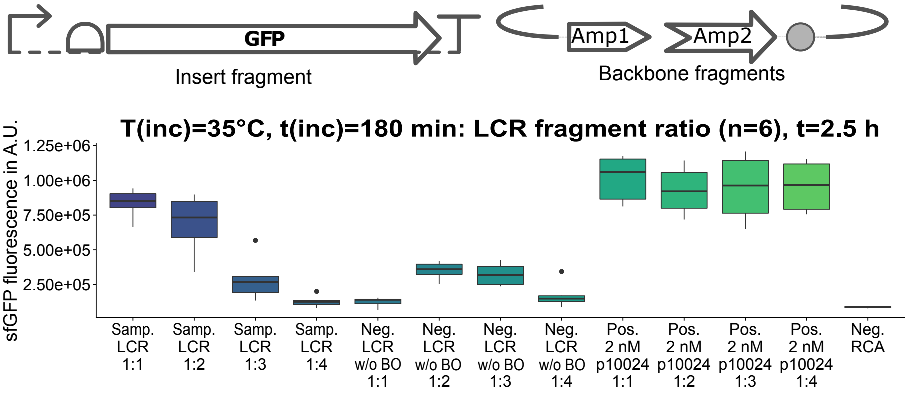

Fig. S 2: Effect of different vector-to-insert ratios (1:1, 1:2, 1:3, 1:4) on the LCR assembly efficiency of a three-part GFP expression construct p10024 (see top schematic), assayed using a subsequent RCA amplification, CFPS and fluorescence measurement of the expression construct. nm T.: nanomolar template

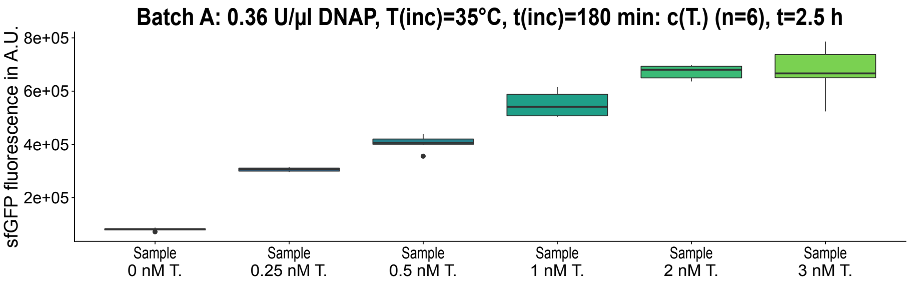

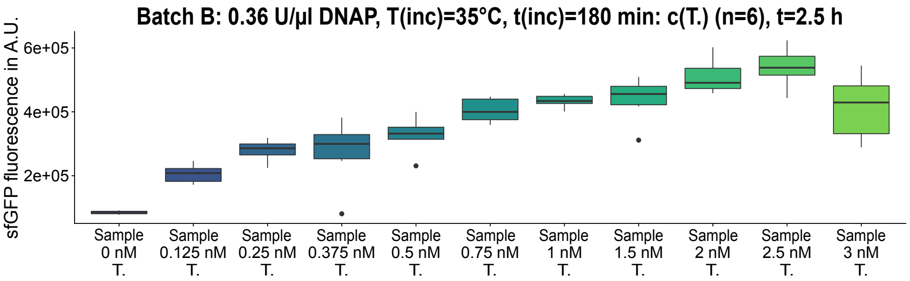

Fig. S 3: Data for batch-to-batch comparison: Effect of different template DNA concentrations in the RCA, assayed using a following CFPS with two different batches and fluorescence measurement of the reporter construct. nm T.: nanomolar template

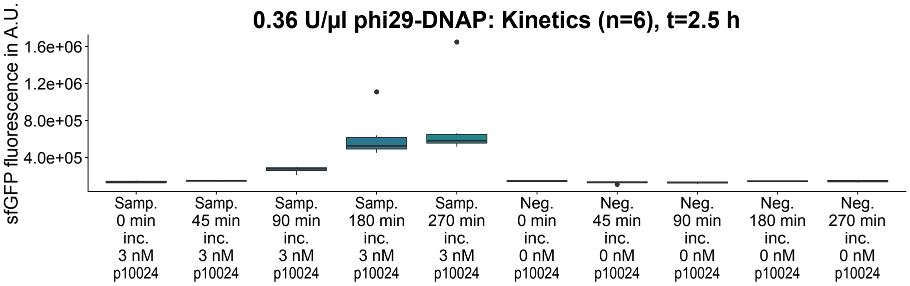

Fig. S 4: Effect of different RCA incubation times of a GFP expression construct (p10024) using a self-made amplfication mix, followed by CFPS, on the sfGFP fluorescence. nm T.: nanomolar template.

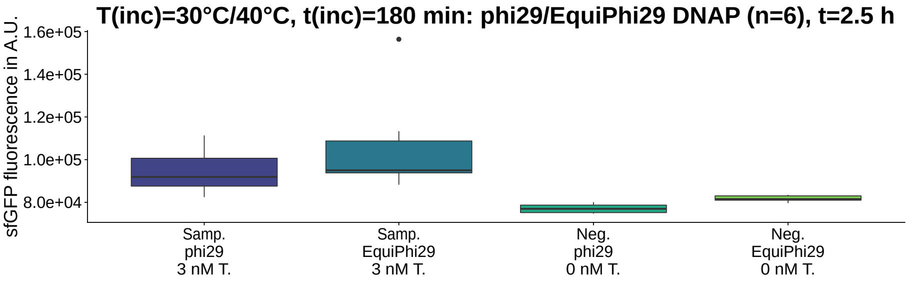

Fig. S 5: Comparison of CFPS for a GFP expression construct with RCA amplification using either phi29- or EquiPhi29™-polymerase. nm T.: nanomolar template.

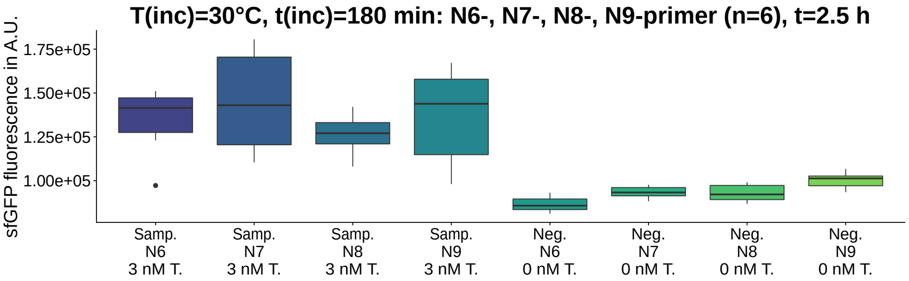

Fig. S 6: Effect of different length random oligos for priming RCA, assayed using a following CFPS and fluorescence measurement of the reporter construct. N6: hexamer; N7: heptamer; N8: octamer; N9: nonamer; nm T.: nanomolar template.

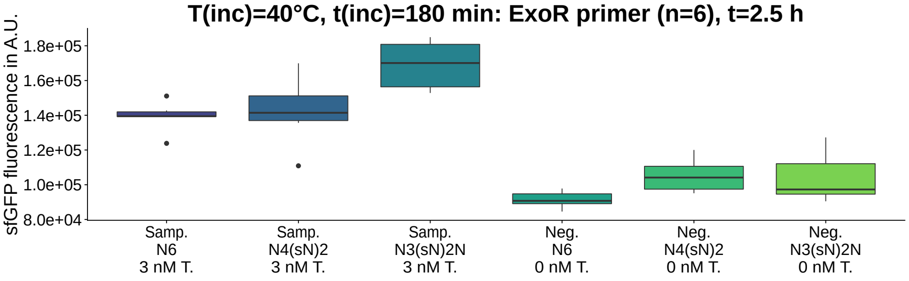

Fig. S 7: Effect of protecting random hexamers through 5'-phosphorothioate modification from exonuclease digestion. N**n**: number of nucleotides; (sN): phosphorothioate modification; nm T.: nanomolar template.

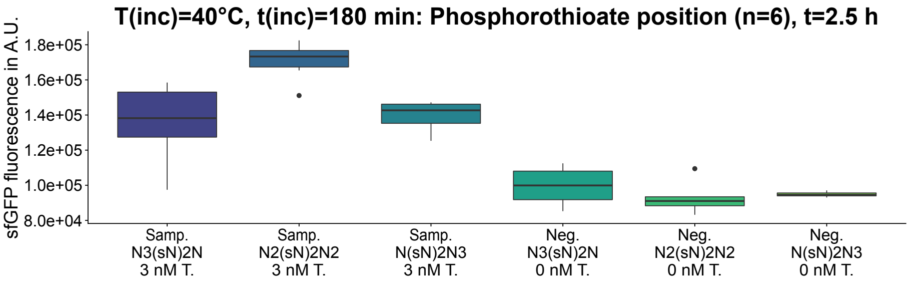

Fig. S 8: Effect of protecting random hexamers through 5'-phosphorothioate modification from exonuclease digestion. N**n**: number of nucleotides; (sN): phosphorothioate modification; nm T.: nanomolar template.

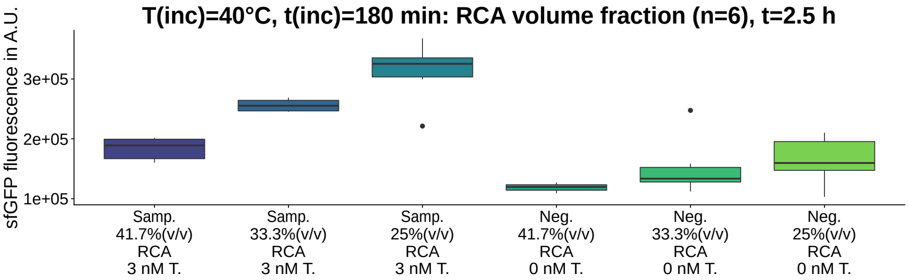

Fig. S 9: RCA volume fraction in the CFPS reaction. nm T.: nanomolar template.

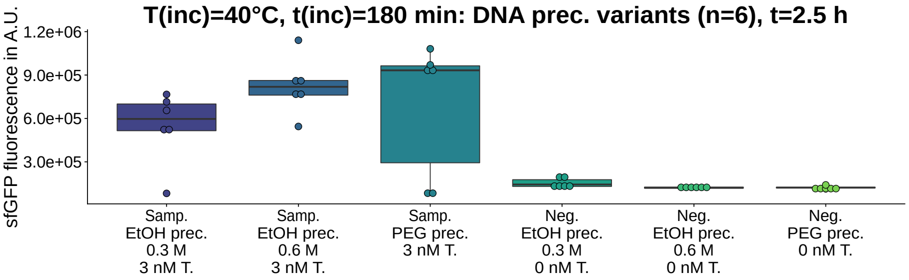

Fig. S 10: Comparision of different precipitation approaches of the RCA reaction. EtOH prec.: ethanol precipitation; PEG prec.: polyethylenglycol precipitation; nm T.: nanomolar template.

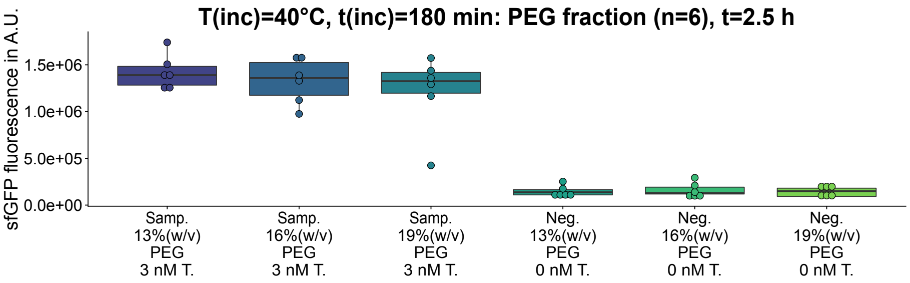

Fig. S 11: Comparision of different concentrations of PEG for precipitation of the RCA reaction. PEG prec.: polyethylenglycol precipitation; nm T.: nanomolar template.

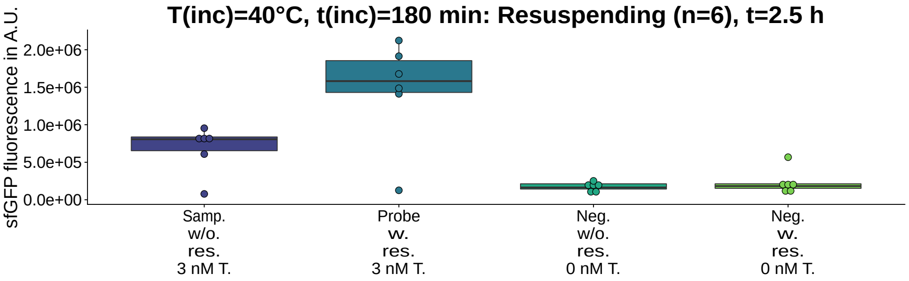

Fig. S 12: Effect of resuspending precipitated DNA after the RCA reaction by inverted centrifugation. nm T.: nanomolar template.

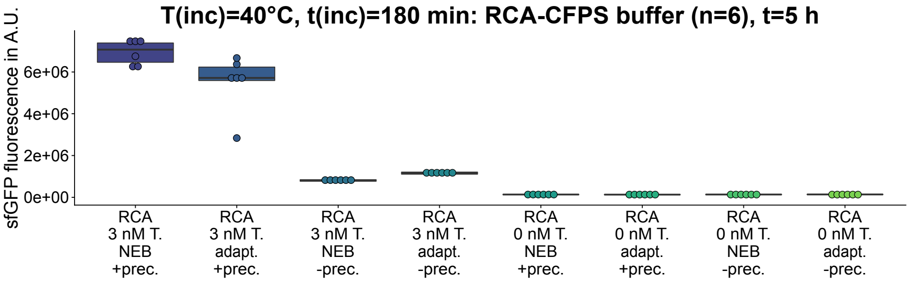

Fig. S 13: Effect of precipitated and unprecipitated RCA-amplified DNA using different buffer systems. nm T.: nanomolar template; NEB: 1x phi29 DNA Polymerase Reaction Buffer by NEB as RCA buffer; adapt: 1x CFPS-adapted RCA buffer as RCA buffer; prec.: precipitated. (Composition of 10x CFPS-adapted RCA buffer: 500 mM Tris base (M=121.14 g/mol), 100 mM magnesium L-glutamate (M=388.61 g/mol), 200 mM L-glutamic acid (M=147.13 g/mol), 100 mM (NH4)2SO4 (M=132.14 g/mol), pH=8.1 (at 22°C) adjusted with 100% acetic acid.)

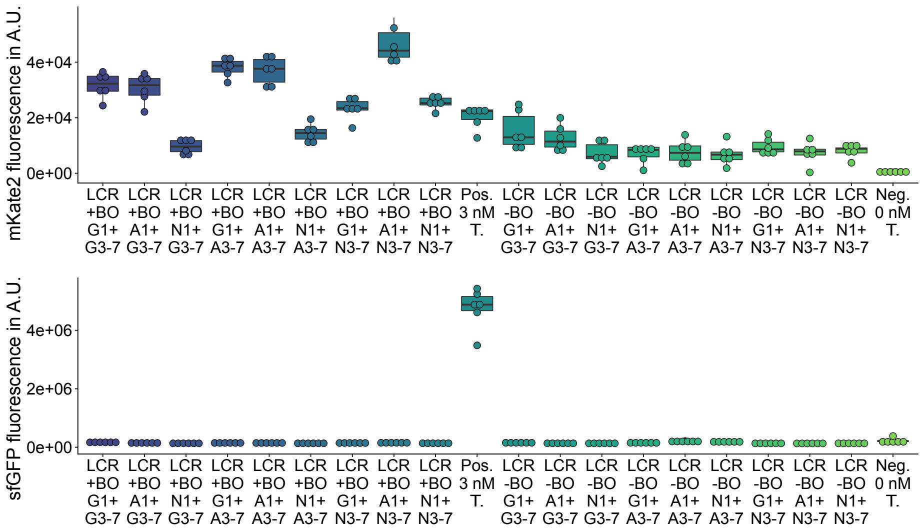

Fig. S 14: Fluorescence measurements of the mKate2 ligation control and GFP assemblies reactions with omitted fragment sequences. *Top:* mKate2 (RFP) measurements of constructs assembled in the presence of bridging oligos (+BO) or the absence of bridging oligos (-BO). *Bottom:* Assembly reactions omitting fragment 2. G*n*:sfGFP fragment *n*; A*n*: mAvicFP1 fragment *n*; N*n*: mNeon-Green fragment *n*; Pos.: 3 nM of the template plasmid used to obtain the fragments for assembly; Neg.: No DNA added.

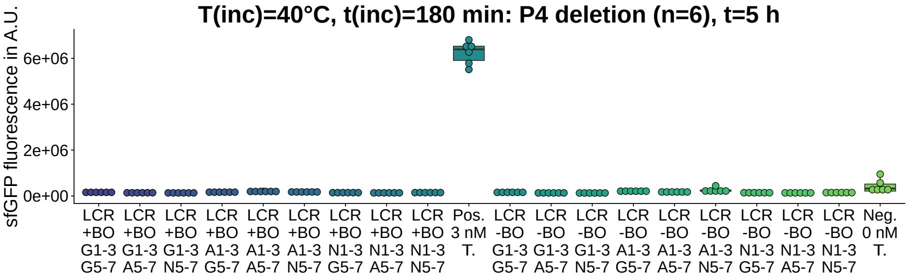

Fig. S 15: Effect of deletion of β-sheet fragment 4 (see Figure 2 in the manuscript) on the GFP fluorescence signal. Gn: sfGFP fragment n; An: mAvicFP1 fragment n; Nn: mNeonGreen fragment n; Pos.: 3 nM of the template plasmid used to obtain the vector for assembly; Neg.: No DNA added.

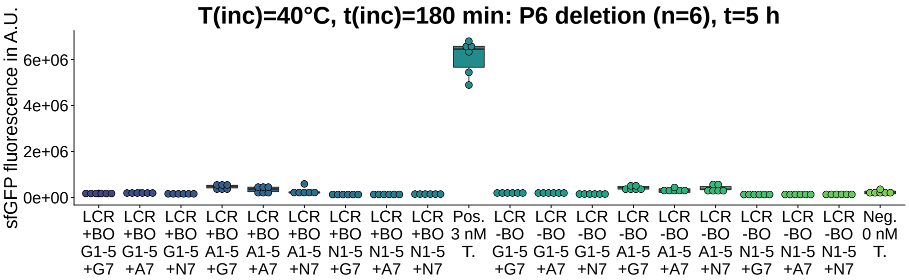

Fig. S 16: Effect of deletion of β-sheet fragment 6 (see Figure 2 in the manuscript) on the GFP fluorescence signal. Gn: sfGFP fragment n; An: mAvicFP1 fragment n; Nn: mNeonGreen fragment n; Pos.: 3 nM of the template plasmid used to obtain the vector for assembly; Neg.: No DNA added.

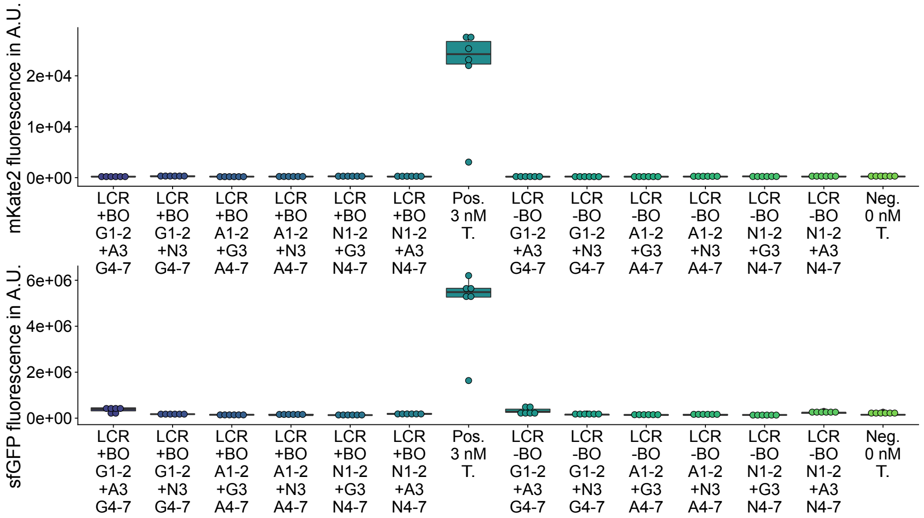

Fig. S 17: GFP- and mKate2-measurements of assemblies with substituted sequences. Gn: sfGFP fragment n; An: mAvicFP1 fragment n; Nn: NeonGreen fragment n; Pos.: 3 nM of the template plasmid used to obtain the fragments for assembly; Neg.: No DNA added.

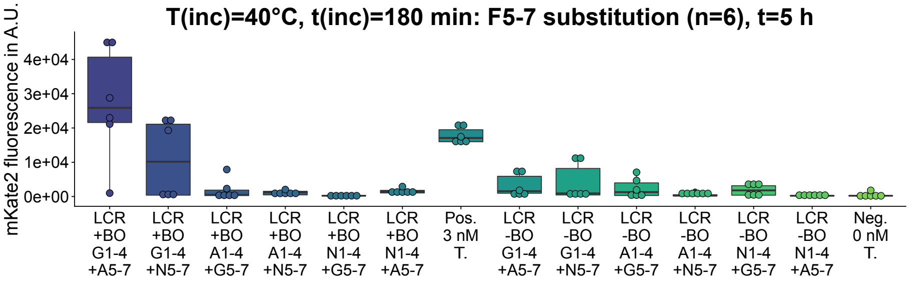

Fig. S 18: Fluorescence measurements of the mKate2 ligation control for *Figure 3: GFP-measurements of assemblies with hybrid sequence*. Gn: sfGFP fragment n; An: mAvicFP1 fragment n; Nn: mNeonGreen fragment n; Pos.: 3 nM of the template plasmid used to obtain the fragments for assembly; Neg.: No DNA added.

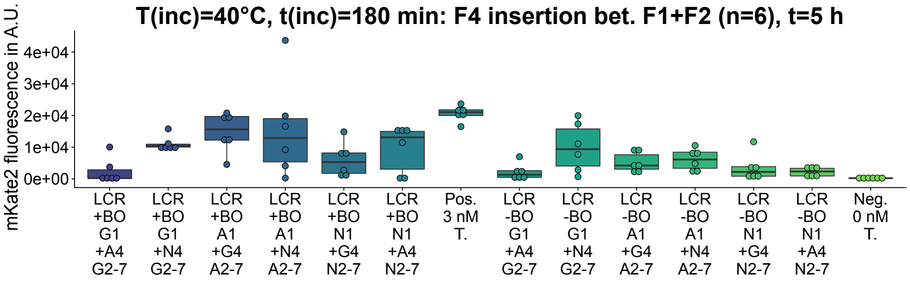

Fig. S 19: Fluorescence measurements of the mKate2 ligation control for F4 *Figure 4 top: GFP-measurements of assemblies with additional sequences inserted*. Gn: sfGFP fragment n; An: mAvicFP1 fragment n; Nn: mNeonGreen fragment n; Pos.: 3 nM of the template plasmid used to obtain the fragments for assembly; Neg.: No DNA added.

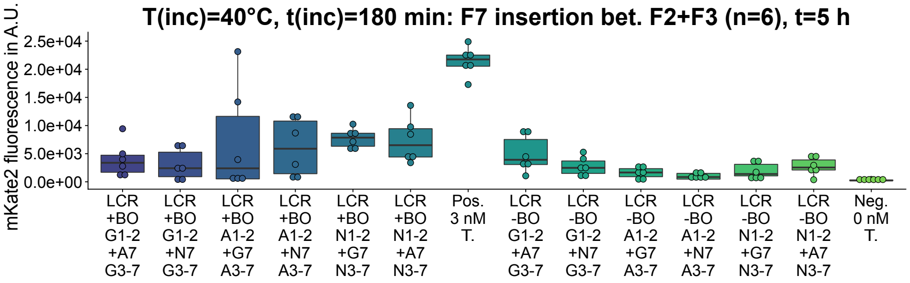

Fig. S 20: Fluorescence measurements of the mKate2 ligation control for F7 *Figure 4 bottom: GFP-measurements of assemblies with additional sequences inserted*. Gn: sfGFP fragment n; An: mAvicFP1 fragment n; Nn: mNeonGreen fragment n; Pos.: 3 nM of the template plasmid used to obtain the fragments for assembly; Neg.: No DNA added.

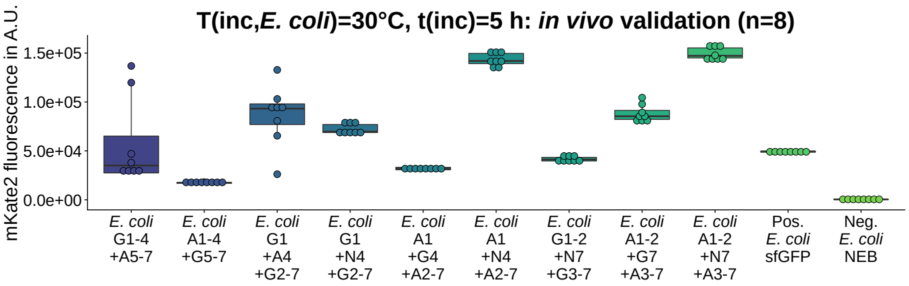

Fig. S 21: Fluorescence measurements of the mKate2 ligation control for *Figure 5, top: GFP-measurements validating constructs previously assembled and screened with HyperXpress.* Gn: sfGFP fragment n; An: mAvicFP1 fragment n; Nn: mNeonGreen fragment n; Pos.: 3 nM of the template plasmid used to obtain the fragments for assembly; Neg.: No DNA added.

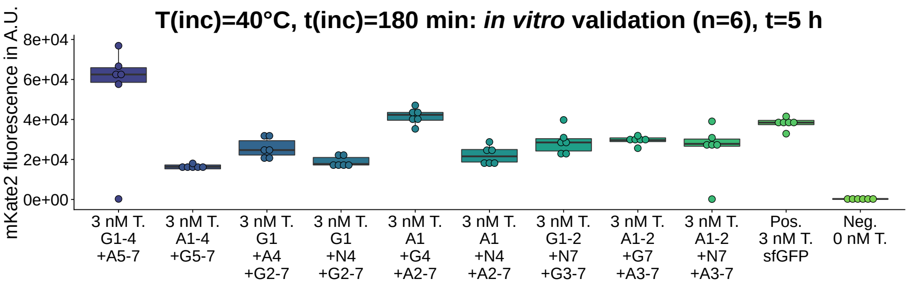

Fig. S 22: Fluorescence measurements of the mKate2 ligation control for *Figure 5, bottom: GFP-measurements validating constructs previously assembled and screened with HyperXpress.* Gn: sfGFP fragment n; An: mAvicFP1 fragment n; Nn: mNeonGreen fragment n; Pos.: 3 nM of the template plasmid used to obtain the fragments for assembly; Neg.: No DNA added.

2.2 Supplemental Table

Table 1: **Bridging oligos used for assemblies. Calculated using custom DIVA-addon. GFPn: sfGFP fragment n; AFn: mAvicFP1 fragment n; NGn: mNeonGreen fragment n**

BO-ID	Assembly of:	Sequence (5′ → 3′)
09073	Vector + GFP1	TTAGCTACTAGAGAAAGAGGAGAAATACTAGA TGAGCAAAGGAGAAGAACTTTTCACTG
09074	GFP1 + GFP2	CAATTCTTGTTGAATTAGATGGTGATGTTAATG GGCACAAATTTTCTGTCCGTG
09075	GFP2 + GFP3	AACTCACCCTTAAATTTATTTGCACTACTGGAA AACTACCTGTTCCGTGGC
09076	GFP3 + GFP4	TATGTACAGGAACGCACTATATCTTTCAAAGAT GACGGGACCTACAAGACG
09077	GFP4 + GFP5	GTTAATCGTATCGAGTTAAAGGGTATTGATTTT AAAGAAGATGGAAACATTCTTGGACACAAA
09078	GFP5 + GFP6	TGTATACATCACGGCAGACAAACAAAAGAATG GAATCAAAGCTAACTTCAAAATT
09079	GFP6 + GFP7	GCAGACCATTATCAACAAAATACTCCAATTGGC GATGGCCCTGTCCTTTTA
09080	GFP7 + Vector	ATGGCATGGATGAGCTCTACAAATAACCTCTAG AAATAATTTTGTTTAACTTTAAGAAGGAGATAT A
09089	Vector + AF1	TTAGCTACTAGAGAAAGAGGAGAAATACTAGA TGTCTAAGGGTGCTGAACTGTTCAAC
09090	AF1 + AF2	TCGAACTGAACGGTGACGTTCATGGTCATAAG TTCTCTGTTCGTGGT
09091	AF2 + AF3	ATCAAGTTCGTTTGCACCACCGGTACCCTGCCT GTTCCTTG
09092	AF3 + AF4	AGAAGCGTACCATCTCTTTCCAGGACGACGGTC ATTACAAGACCC
09093	AF4 + AF5	ATCGAACTGAAGGGTATCGACTTCAAGGAAGA CGGTAACATCCTGG
09094	AF5 + AF6	TTTACGTTCTGTCTGACAAGGCTAACAACGGTA TCAAGGTTAACTTCAAG
09095	AF6 + AF7	CATGACCAGCAGAACATCCCTATCGGTGACGGT CCTGTTCTGC
09096	AF7 + Vector	CCATGGTATGGACGAACTGTACAAGTAACCTCT AGAAATAATTTTGTTTAACTTTAAGAAGGAGAT ATA
09097	Vector + NG1	TTAGCTACTAGAGAAAGAGGAGAAATACTAGA TGGTGTCCAAGGGAGAGGAGGAT
09098	NG1 + NG2	ATGAACTTCATATCTTTGGTTCGATCAACGGAG TGGACTTTGATATGGTTGGT

continued on next page

BO-ID	Assembly of:	Sequence (5′ → 3′)
09099	NG2 + NG3	GGTATGAAGAGTTGAATCTTAAATCGACAAAA GGGGATCTTCAATTCAGCCCT
09100	NG3 + NG4	CGCACCATGCAGTTCGAGGACGGAGCATCTCTT ACTGTTAACTAC
09101	NG4 + NG5	GCTCAGGTTAAAGGAACGGGATTTCCCGCTGA CGGACCC
09102	NG5 + NG6	GTGTCGCAGTAAGAAAACTTATCCTAACGACAA AACGATTATCTCGACGTTCAAA
09103	NG6 + NG7	GCACGACGTACACCTTTGCCAAACCGATGGCG GCTAACTAT
09104	NG7 + Vector	CGTGATGGGGATGGATGAGTTATATAAATAAC CTCTAGAAATAATTTTGTTTAACTTTAAGAAGG AGATATA
09105	GFP1 + GFP3	CAATTCTTGTTGAATTAGATGGTGATGTTAATG GAAAACTACCTGTTCCGTGGC
09107	GFP1 + AF3	CAATTCTTGTTGAATTAGATGGTGATGTTAATG GTACCCTGCCTGTTCCTTG
09108	GFP1 + NG3	CAATTCTTGTTGAATTAGATGGTGATGTTAATG GGGATCTTCAATTCAGCCCT
09113	AF1 + GFP3	TCGAACTGAACGGTGACGTTCATGGAAAACTA CCTGTTCCGTGGC
09115	AF1 + AF3	TCGAACTGAACGGTGACGTTCATGGTACCCTGC CTGTTCCTTG
09116	AF1 + NG3	TCGAACTGAACGGTGACGTTCATGGGGATCTT CAATTCAGCCCT
09117	NG1 + GFP3	ATGAACTTCATATCTTTGGTTCGATCAACGGAA AACTACCTGTTCCGTGGC
09119	NG1 + AF3	ATGAACTTCATATCTTTGGTTCGATCAACGGTA CCCTGCCTGTTCCTTG
09120	NG1 + NG3	ATGAACTTCATATCTTTGGTTCGATCAACGGGG ATCTTCAATTCAGCCCT
09126	GFP3 + GFP5	TATGTACAGGAACGCACTATATCTTTCAAAAAA GAAGATGGAAACATTCTTGGACACAAA
09128	GFP3 + AF5	TATGTACAGGAACGCACTATATCTTTCAAAAAG GAAGACGGTAACATCCTGG
09129	GFP3 + NG5	TATGTACAGGAACGCACTATATCTTTCAAACCC GCTGACGGACCC
09134	AF3 + GFP5	AGAAGCGTACCATCTCTTTCCAGAAAGAAGATG GAAACATTCTTGGACACAAA
09136	AF3 + AF5	AGAAGCGTACCATCTCTTTCCAGAAGGAAGAC GGTAACATCCTGG
09137	AF3 + NG5	AGAAGCGTACCATCTCTTTCCAGCCCGCTGACG GACCC
09138	NG3 + GFP5	CGCACCATGCAGTTCGAGAAAGAAGATGGAAA CATTCTTGGACACAAA

continued on next page

BO-ID	Assembly of:	Sequence ($5' \rightarrow 3'$)
09140	NG3 + AF5	CGCACCATGCAGTTCGAGAAGGAAGACGGTAACATCCTGG
09141	NG3 + NG5	CGCACCATGCAGTTCGAGCCCGCTGACGGACCC
09142	GFP5 + GFP7	TGTATACATCACGGCAGACAAACAAGGCGATGGCCCTGTCCTTTTA
09144	GFP5 + AF7	TGTATACATCACGGCAGACAAACAAGGTGACGGTCCTGTTCTGC
09145	GFP5 + NG7	TGTATACATCACGGCAGACAAACAAAAACCGATGGCGGCTAACTAT
09150	AF5 + GFP7	TTTACGTTCTGTCTGACAAGGCTGGCGATGGCCCTGTCCTTTTA
09152	AF5 + AF7	TTTACGTTCTGTCTGACAAGGCTGGTGACGGTCCTGTTCTGC
09153	AF5 + NG7	TTTACGTTCTGTCTGACAAGGCTAAACCGATGGCGGCTAACTAT
09154	NG5 + GFP7	GTGTCGCAGTAAGAAAACTTATCCTAACGGCGATGGCCCTGTCCTTTTA
09156	NG5 + AF7	GTGTCGCAGTAAGAAAACTTATCCTAACGGTGACGGTCCTGTTCTGC
09157	NG5 + NG7	GTGTCGCAGTAAGAAAACTTATCCTAACAAACCGATGGCGGCTAACTAT
09173	GFP2 + AF7	AACTCACCCTTAAATTTATTTGCACTACTGGTGACGGTCCTGTTCTGC
09174	GFP2 + NG7	AACTCACCCTTAAATTTATTTGCACTACTAAACCGATGGCGGCTAACTAT
09175	AF2 + GFP7	ATCAAGTTCGTTTGCACCACCGGCGATGGCCCTGTCCTTTTA
09176	AF2 + NG7	ATCAAGTTCGTTTGCACCACCAAACCGATGGCGGCTAACTAT
09177	NG2 + GFP7	GGTATGAAGAGTTGAATCTTAAATCGACAAAAGGCGATGGCCCTGTCCTTTTA
09178	NG2 + AF7	GGTATGAAGAGTTGAATCTTAAATCGACAAAAGGTGACGGTCCTGTTCTGC
09179	GFP7 + AF3	ATGGCATGGATGAGCTCTACAAAGGTACCCTGCCTGTTCCTTG
09180	GFP7 + NG3	ATGGCATGGATGAGCTCTACAAAGGGGATCTTCAATTCAGCCCT
09181	AF7 + GFP3	CCATGGTATGGACGAACTGTACAAGGGAAAACTACCTGTTCCGTGGC
09182	AF7 + NG3	CCATGGTATGGACGAACTGTACAAGGGGGATCTTCAATTCAGCCCT
09183	NG7 + GFP3	CGTGATGGGGATGGATGAGTTATATAAAGGAAAACTACCTGTTCCGTGGC
09184	NG7 + AF3	CGTGATGGGGATGGATGAGTTATATAAAGGTACCCTGCCTGTTCCTTG

continued on next page

BO-ID	Assembly of:	Sequence (5′ → 3′)
09195	GFP1 + AF4	CCAATTCTTGTTGAATTAGATGGTGATGTTAAT GACGACGGTCATTACAAGACCC
09196	GFP1 + NG4	CCAATTCTTGTTGAATTAGATGGTGATGTTAAT GACGGAGCATCTCTTACTGTTAACTAC
09197	AF1 + GFP4	TCGAACTGAACGGTGACGTTCATGATGACGGG ACCTACAAGACG
09198	AF1 + NG4	TCGAACTGAACGGTGACGTTCATGACGGAGCA TCTCTTACTGTTAACTAC
09199	NG1 + GFP4	ATGAACTTCATATCTTTGGTTCGATCAACGATG ACGGGACCTACAAGACG
09200	NG1 + AF4	ATGAACTTCATATCTTTGGTTCGATCAACGACG ACGGTCATTACAAGACCC
09201	GFP4 + AF2	GTTAATCGTATCGAGTTAAAGGGTATTGATTTT GGTCATAAGTTCTCTGTTCGTGGT
09202	GFP4 + NG2	GTTAATCGTATCGAGTTAAAGGGTATTGATTTT GGAGTGGACTTTGATATGGTTGGT
09203	AF4 + GFP2	ATCGAACTGAAGGGTATCGACTTCGGGCACAA ATTTTCTGTCCGTG
09204	AF4 + NG2	ATCGAACTGAAGGGTATCGACTTCGGAGTGGA CTTTGATATGGTTGGT
09205	NG4 + GFP2	GCTCAGGTTAAAGGAACGGGATTTGGGCACAA ATTTTCTGTCCGTG
09206	NG4 + AF2	GCTCAGGTTAAAGGAACGGGATTTGGTCATAA GTTCTCTGTTCGTGGT
09207	GFP2 + AF3	AACTCACCCTTAAATTTATTTGCACTACTGGTA CCCTGCCTGTTCCTTG
09208	GFP2 + NG3	AACTCACCCTTAAATTTATTTGCACTACTGGGG ATCTTCAATTCAGCCCT
09209	AF2 + GFP3	ATCAAGTTCGTTTGCACCACCGGAAAACTACCT GTTCCGTGGC
09210	AF2 + NG3	ATCAAGTTCGTTTGCACCACCGGGGATCTTCAA TTCAGCCCT
09211	NG2 + GFP3	GGTATGAAGAGTTGAATCTTAAATCGACAAAA GGAAAACTACCTGTTCCGTGGC
09212	NG2 + AF3	GGTATGAAGAGTTGAATCTTAAATCGACAAAA GGTACCCTGCCTGTTCCTTG
09213	GFP3 + AF4	TATGTACAGGAACGCACTATATCTTTCAAAGAC GACGGTCATTACAAGACCC
09214	GFP3 + NG4	TATGTACAGGAACGCACTATATCTTTCAAAGAC GGAGCATCTCTTACTGTTAACTAC
09215	AF3 + GFP4	AGAAGCGTACCATCTCTTTCCAGGATGACGGG ACCTACAAGACG
09216	AF3 + NG4	AGAAGCGTACCATCTCTTTCCAGGACGGAGCAT CTCTTACTGTTAACTAC
09217	NG3 + GFP4	CGCACCATGCAGTTCGAGGATGACGGGACCTA CAAGACG

continued on next page

BO-ID	Assembly of:	Sequence ($5' \rightarrow 3'$)
09218	NG3 + AF4	CGCACCATGCAGTTCGAGGACGACGGTCATTACAAGACCC
09219	GFP4 + AF5	GTTAATCGTATCGAGTTAAAGGGTATTGATTTTAAGGAAGACGGTAACATCCTGG
09220	GFP4 + NG5	GTTAATCGTATCGAGTTAAAGGGTATTGATTTTCCCGCTGACGGACCC
09221	AF4 + GFP5	ATCGAACTGAAGGGTATCGACTTCAAAGAAGATGGAAACATTCTTGGACACAAA
09222	AF4 + NG5	ATCGAACTGAAGGGTATCGACTTCCCCGCTGACGGACCC
09223	NG4 + GFP5	GCTCAGGTTAAAGGAACGGGATTTAAAGAAGATGGAAACATTCTTGGACACAAA
09224	NG4 + AF5	GCTCAGGTTAAAGGAACGGGATTTAAGGAAGACGGTAACATCCTGG
		End of bridging oligo table

Table 2: **Primer used for part amplification. GFP*n*: sfGFP fragment *n*; AF*n*: mAvicFP1 fragment *n*; NG*n*: mNeonGreen fragment *n*; s: position of 5'-phosphorothioate modification in random nucleotide oligos.**

Primer-ID	Used for	Sequence ($5' \rightarrow 3'$)
09005	fw primer for vector amplification	TAACCTCTAGAAATAATTTTGTTTAAC
09006	rev primer for vector amplification	CATCTAGTATTTCTCCTCTTTC
09007	random primer for RCA	NNNNNNNNN
09012	fw primer for GFP1 amplification	AGCAAAGGAGAAGAACTTTTC
09013	rev primer for GFP1 amplification	ATTAACATCACCATCTAATTCAACA
09014	fw primer for GFP2 amplification	GGGCACAAATTTTCTGTCC
09015	rev primer for GFP2 amplification	AGTAGTGCAAATAAATTTAAGGGT
09016	fw primer for GFP3 amplification	GGAAAACTACCTGTTCCGT
09017	rev primer for GFP3 amplification	TTTGAAAGATATAGTGCGTTCC
09018	fw primer for GFP4 amplification	GATGACGGGACCTACAAG
09019	rev primer for GFP4 amplification	AAAATCAATACCCTTTAACTCGA
09020	fw primer for GFP5 amplification	AAAGAAGATGGAAACATTCTTGGACA
09021	rev primer for GFP5 amplification	TTGTTTGTCTGCCGTGATGTATAC
09022	fw primer for GFP6 amplification	AAGAATGGAATCAAAGCTAACT
09023	rev primer for GFP6 amplification	AATTGGAGTATTTTGTTGATAATGG
09024	fw primer for GFP7 amplification	GGCGATGGCCCTGTCCTTTTA
09025	rev primer for GFP7 amplification	TTTGTAGAGCTCATCCATGCCATGT
09040	fw primer for NG1 amplification	GTGTCCAAGGGAGAGGA
09041	rev primer for NG1 amplification	GTTGATCGAACCAAAGATATGAA
09042	fw primer for NG2 amplification	GGAGTGGACTTTGATATGGTT
09043	rev primer for NG2 amplification	TTTTGTCGATTTAAGATTCAACTC

continued on next page

Primer-ID	Used for	Sequence ($5' \rightarrow 3'$)
09044	fw primer for NG3 amplification	GGGGATCTTCAATTCAGC
09045	rev primer for NG3 amplification	CTCGAACTGCATGGT
09046	fw primer for NG4 amplification	GACGGAGCATCTCTTACTGTTA
09047	rev primer for NG4 amplification	AAATCCCGTTCCTTTAACCTGA
09048	fw primer for NG5 amplification	CCCGCTGACGGACCCGTAATGACT
09049	rev primer for NG5 amplification	GTTAGGATAAGTTTTCTTACTGCGACACC AGTCG
09050	fw primer for NG6 amplification	GACAAAACGATTATCTCGACGTTCAA
09051	rev primer for NG6 amplification	GGCAAAGGTGTACGTCGTG
09052	fw primer for NG7 amplification	AAACCGATGGCGGCTAACTATCTGAAGA
09053	rev primer for NG7 amplification	TTTATATAACTCATCCATCCCCATCACGT CCG
09054	fw primer for AF1 amplification	TCTAAGGGTGCTGAACTGTTCAACGGTA TCG
09055	rev primer for AF1 amplification	ATGAACGTCACCGTTCAGTTCGATCAGG ATAGGA
09056	fw primer for AF2 amplification	GGTCATAAGTTCTCTGTTCGTGGT
09057	rev primer for AF2 amplification	GGTGGTGCAAACGAACTTGATT
09058	fw primer for AF3 amplification	GGTACCCTGCCTGTTCC
09059	rev primer for AF3 amplification	CTGGAAAGAGATGGTACGCT
09060	fw primer for AF4 amplification	GACGACGGTCATTACAAGAC
09061	rev primer for AF4 amplification	GAAGTCGATACCCTTCAGTTC
09062	fw primer for AF5 amplification	AAGGAAGACGGTAACATCC
09063	rev primer for AF5 amplification	AGCCTTGTCAGACAGAAC
09064	fw primer for AF6 amplification	AACAACGGTATCAAGGTTAAC
09065	rev primer for AF6 amplification	GATAGGGATGTTCTGCTGGT
09066	fw primer for AF7 amplification	GGTGACGGTCCTGTTCT
09067	rev primer for AF7 amplification	CTTGTACAGTTCGTCCATACC
09071	random primer for RCA	NNNNNNN
09072	random primer for RCA	NNNNNNNN
09185	random primer for RCA	NNNNsNsN
09186	random primer for RCA	NNNsNsNN
09187	random primer for RCA	NNsNsNNN
09188	random primer for RCA	NsNsNNNN
09193	fw primer for AF ORF amplification	TCTAAGGGTGCTGAACTGTT

End of primer oligo table

Table 3: *E. coli* strains used and generated in this study.

Strain ID	Organism	Plasmid	Resistence	Origin
S10037	E. coli	p10024	Amp	AG Koeppl, TU Darmstadt
S10036	E. coli Rosetta EL22	-	Chl, Kan	AG Koeppl, TU Darmstadt
S09002	E. coli NEB10-beta	p09002	Kan	AG Stein,
S09004	E. coli DH5α	p09004	Amp	Addgene #129509
S09005	E. coli NEB10-beta	p09005	Amp	AG Kabisch, TU Darmstadt
S09007	E. coli NEB10-beta	p09007	Amp	AG Kabisch, TU Darmstadt
S09008	E. coli NEB10-beta	p09008	Amp	AG Kabisch, TU Darmstadt (deposited at Addgene as #173715)
S09009	E. coli NEB10-beta	p09009	Amp	AG Kabisch, TU Darmstadt
S09010	E. coli NEB10-beta	p09010	Amp	AG Kabisch, TU Darmstadt
S09011	E. coli NEB10-beta	p09011	Amp	AG Kabisch, TU Darmstadt
S09012	E. coli NEB10-beta	p09012	Amp	AG Kabisch, TU Darmstadt
S09013	E. coli NEB10-beta	p09013	Amp	AG Kabisch, TU Darmstadt
S09014	E. coli NEB10-beta	p09014	Amp	AG Kabisch, TU Darmstadt
S09015	E. coli NEB10-beta	p09015	Amp	AG Kabisch, TU Darmstadt

3 Genbank files of Plasmids

3.0.1 p10024

```
LOCUS       Exported               2918 bp DNA     circular SYN 22-OCT-2020
DEFINITION  p10024
ACCESSION
VERSION     .
KEYWORDS    .
SOURCE      synthetic DNA construct
  ORGANISM  synthetic DNA construct
REFERENCE   1  (bases 1 to 2918)
FEATURES             Location/Qualifiers
     source          1..2918
                     /organism="synthetic DNA construct"
                     /mol_type="other DNA"
     CDS             complement(join(1..497,2756..2918))
                     /standard_name="AmpR"
                     /label=AmpR
     primer_bind     214..261
                     /standard_name="10449_LCR_BB1-BB2_fw"
                     /label=10449_LCR_BB1-BB2_fw
     misc_feature    complement(219..236)
                     /standard_name="10444_BB1_rv"
                     /label=10444_BB1_rv
                     /note="Geneious type: primer_bind_reverse"
     primer_bind     237..255
                     /standard_name="10445_BB2_fw"
                     /label=10445_BB2_fw
     misc_feature    complement(533..538)
                     /standard_name="BamHI-BglII\Scar(7)"
                     /label=BamHI-BglII Scar(7)
     misc_feature    complement(533..538)
                     /standard_name="BamHI-BglII\Scar"
                     /label=BamHI-BglII Scar
     misc_feature    550..555
                     /standard_name="BamHI-BglII\Scar(3)"
                     /label=BamHI-BglII Scar(3)
```

```
primer_bind     908..964
                /standard_name="10447_LCR_BB2-tGFP_fw"
                /label=10447_LCR_BB2-tGFP_fw
misc_feature    complement(913..934)
                /standard_name="10446_BB2_rv"
                /label=10446_BB2_rv
                /note="Geneious type: primer_bind_reverse"
primer_bind     935..958
                /standard_name="10441_tGFP_fw"
                /label=10441_tGFP_fw
misc_feature    951..985
                /standard_name="Promoter-J23119(SpeI)"
                /label=Promoter-J23119(SpeI)
misc_feature    982..985
                /standard_name="BbsI"
                /label=BbsI
RBS             1014..1021
CDS             1030..1746
                /standard_name="sfGFP"
                /label=sfGFP
CDS             1033..1098
                /codon_start=1
                /label=GFP1_(ohne AUG)
                /translation="SKGEELFTGVVPILVELDGDVN"
primer_bind     1033..1053
                /label=09012_GFP1_fw
primer_bind     complement(1074..1098)
                /label=09013_GFP1_rev
CDS             1099..1179
                /codon_start=1
                /label=GFP2
                /translation="GHKFSVRGEGEGDATNGKLTLKFICTT"
primer_bind     1099..1117
                /label=09014_GFP2_fw
primer_bind     complement(1156..1179)
                /label=09015_GFP2_rev
CDS             1180..1332
                /codon_start=1
                /label=GFP3
                /translation="GKLPVPWPTLVTTLTYGVQCFSRYPDHMKRHDFFKSAMPEGYVQE
                RTISFK"
primer_bind     1180..1198
                /label=09016_GFP3_fw
primer_bind     complement(1311..1332)
                /label=09017_GFP3_rev
CDS             1333..1419
                /codon_start=1
                /label=GFP4
                /translation="DDGTYKTRAEVKFEGDTLVNRIELKGIDF"
primer_bind     1333..1350
                /label=09018_GFP4_fw
misc_feature    complement(1347..1364)
                /standard_name="10296_sGFP_rv"
                /label=10296_sGFP_rv
                /note="Geneious type: primer_bind_reverse"
primer_bind     1365..1387
                /standard_name="10295_sGFP_fw"
                /label=10295_sGFP_fw
primer_bind     complement(1397..1419)
                /label=09019_GFP4_rev
CDS             1420..1500
                /codon_start=1
                /label=GFP5
                /translation="KEDGNILGHKLEYNFNSHNVYITADKQ"
primer_bind     1420..1445
                /label=09020_GFP5_fw
primer_bind     complement(1477..1500)
                /label=09021_GFP5_rev
```

```
     CDS             1501..1593
                     /codon_start=1
                     /label=GFP6
                     /translation="KNGIKANFKIRHNVEDGSVQLADHYQQNTPI"
     primer_bind     1501..1522
                     /label=09022_GFP6_fw
     primer_bind     complement(1569..1593)
                     /label=09023_GFP6_rev
     CDS             1594..1743
                     /codon_start=1
                     /label=GFP7_(ohne UAA)
                     /translation="GDGPVLLPDNHYLSTQSVLSKDPNEKRDHMVLLEFVTAAGITHGM
                     DELYK"
     primer_bind     1594..1614
                     /label=09024_GFP7_fw
     primer_bind     complement(1719..1743)
                     /label=09025_GFP7_rev
     3'UTR           1747..1760
                     /label=3'-UTR
     misc_feature    complement(1755..1760)
                     /standard_name="BamHI-BglII\Scar(4)"
                     /label=BamHI-BglII Scar(4)
     misc_feature    1755..1758
                     /standard_name="BbsI"
                     /label=BbsI
     misc_feature    1761..1790
                     /standard_name="t500"
                     /label=t500
     primer_bind     1787..1831
                     /standard_name="10448_LCR_tGFP-BB1_fw"
                     /label=10448_LCR_tGFP-BB1_fw
     misc_feature    complement(1791..1812)
                     /standard_name="10442_tGFP_rv"
                     /label=10442_tGFP_rv
                     /note="Geneious type: primer_bind_reverse"
     primer_bind     1813..1827
                     /standard_name="10443_BB1_fw"
                     /label=10443_BB1_fw
     rep_origin      1976..2658
     misc_feature    complement(2574..2579)
                     /standard_name="BamHI-BglII\Scar(5)"
                     /label=BamHI-BglII Scar(5)
     misc_feature    2585..2590
                     /standard_name="BamHI-BglII\Scar(1)"
                     /label=BamHI-BglII Scar(1)
     misc_feature    complement(2671..2676)
                     /standard_name="BamHI-BglII\Scar(6)"
                     /label=BamHI-BglII Scar(6)
     misc_feature    2683..2688
                     /standard_name="BamHI-BglII\Scar(2)"
                     /label=BamHI-BglII Scar(2)
ORIGIN
        1 ttatcagcaa taaaccagcc agccggaagg gccgagcgca gaagtggtcc tgcaacttta
       61 tccgcctcca tccagtctat taattgttgc cgggaagcta gagtaagtag ttcgccagtt
      121 aatagtttgc gcaacgttgt tgccattgct acaggcatcg tggtgtcacg ctcgtcgttt
      181 ggtatggctt cattcagctc cggttcccaa cgatcaaggc gagttacatg atcccccatg
      241 ttgtgcaaaa aagcggttag ctccttcggt cctccgatcg ttgtcagaag taagttggcc
      301 gcagtgttat cactcatggt tatggcagca ctgcataatt ctcttactgt catgccatcc
      361 gtaagatgct tttctgtgac tggtgagtac tcaaccaagt cattctgaga atagtgtatg
      421 cggcgaccga gttgctcttg cccggcgtca atacgggata ataccgcgcc acatagcaga
      481 actttaaaag tgctcatcat tggaaaacgt tcttcggggc gaaaactctc aaggatctta
      541 ccgctgttga gatccagttc gatgtaaccc actcgtgcac ccaactgatc ttcagcatct
      601 tttactttca ccagcgtttc tgggtgagca aaaacaggaa ggcaaaatgc cgcaaaaaag
      661 ggaataaggg cgacacggaa atgttgaata ctcatactct tcctttttca atattattga
      721 agcatttatc agggttattg tctcatgagc ggatacatat ttgaatgtat ttagaaaaat
      781 aaacaaatag gggttccgcg cacatttccc cgaaaagtgc cacctgacgt ctaagaaacc
      841 attattatca tgacattaac ctataaaaat aggcgtatca cgaggcagaa tttcagataa
      901 aaaaaatcct tagctttcgc taaggatgat ttctggaatt ctaaagatct ttgacagcta
```

```
      961 gctcagtcct aggtataata ctagtatgtc ttcggatctt agctactaga gaaagaggag
     1021 aaatactaga tgagcaaagg agaagaactt ttcactggag ttgtcccaat tcttgttgaa
     1081 ttagatggtg atgttaatgg gcacaaattt tctgtccgtg gagagggtga aggtgatgct
     1141 acaaacggaa aactcaccct taaatttatt tgcactactg gaaaactacc tgttccgtgg
     1201 ccaacacttg tcactactct gacctatggt gttcaatgct tttcccgtta tccggatcac
     1261 atgaaacggc atgacttttt caagagtgcc atgcccgaag gttatgtaca ggaacgcact
     1321 atatctttca aagatgacgg gacctacaag acgcgtgctg aagtcaagtt tgaaggtgat
     1381 acccttgtta atcgtatcga gttaaagggt attgatttta aagaagatgg aaacattctt
     1441 ggacacaaac tcgagtacaa ctttaactca cacaatgtat acatcacggc agacaaacaa
     1501 aagaatggaa tcaaagctaa cttcaaaatt cgccacaacg ttgaagatgg ttccgttcaa
     1561 ctagcagacc attatcaaca aaatactcca attggcgatg gccctgtcct tttaccagac
     1621 aaccattacc tgtcgacaca atctgtcctt tcgaaagatc ccaacgaaaa gcgtgaccac
     1681 atggtccttc ttgagtttgt aactgctgct gggattacac atggcatgga tgagctctac
     1741 aaataagaag acaaggatct caaagcccgc cgaaaggcgg gcttttttt ggatccttac
     1801 tcgagtctag actgcaggct tcctcgctca ctgactcgct gcgctcggtc gttcggctgc
     1861 ggcgagcggt atcagctcac tcaaaggcgg taatacggtt atccacagaa tcaggggata
     1921 acgcaggaaa gaacatgtga gcaaaaggcc agcaaaaggc caggaaccgt aaaaaggccg
     1981 cgttgctggc gtttttccac aggctccgcc cccctgacga gcatcacaaa aatcgacgct
     2041 caagtcagag gtggcgaaac ccgacaggac tataaagata ccaggcgttt ccccctggaa
     2101 gctccctcgt gcgctctcct gttccgaccc tgccgcttac cggatacctg tccgcctttc
     2161 tcccttcggg aagcgtggcg ctttctcata gctcacgctg taggtatctc agttcggtgt
     2221 aggtcgttcg ctccaagctg ggctgtgtgc acgaaccccc cgttcagccc gaccgctgcg
     2281 ccttatccgg taactatcgt cttgagtcca acccggtaag acacgactta tcgccactgg
     2341 cagcagccac tggtaacagg attagcagag cgaggtatgt aggcggtgct acagagttct
     2401 tgaagtggtg gcctaactac ggctacacta gaagaacagt atttggtatc tgcgctctgc
     2461 tgaagccagt taccttcgga aaaagagttg gtagctcttg atccggcaaa caaaccaccg
     2521 ctggtagcgg tggttttttt gtttgcaagc agcagattac gcgcagaaaa aaaggatctc
     2581 aagaagatcc tttgatcttt tctacggggt ctgacgctca gtggaacgaa aactcacgtt
     2641 aagggatttt ggtcatgaga ttatcaaaaa ggatcttcac ctagatcctt ttaaattaaa
     2701 aatgaagttt taaatcaatc taaagtatat atgagtaaac ttggtctgac agttaccaat
     2761 gcttaatcag tgaggcacct atctcagcga tctgtctatt tcgttcatcc atagttgcct
     2821 gactccccgt cgtgtagata actacgatac gggagggctt accatctggc cccagtgctg
     2881 caatgatacc gcgagaccca cgctcaccgg ctccagat
//
```

3.0.2 p09002

```
LOCUS       Exported               2700 bp DNA     circular SYN 19-OCT-2020
DEFINITION  p09002
ACCESSION   .
VERSION     .
KEYWORDS    .
SOURCE      synthetic DNA construct
  ORGANISM  synthetic DNA construct
REFERENCE   1  (bases 1 to 2700)
FEATURES             Location/Qualifiers
     source          1..2700
                     /organism="synthetic DNA construct"
                     /mol_type="other DNA"
     primer_bind     1..22
                     /label=control fw on bb
     primer_bind     complement(29..50)
                     /label=amp bb gblock2 rev
     primer_bind     complement(171..194)
                     /label=pFD amplify rv
     primer_bind     complement(178..194)
                     /label=oligo pFD mut 2
     CDS             195..899
                     /codon_start=1
                     /label=mNeonGreen
                     /translation="VSKGEEDNMASLPATHELHIFGSINGVDFDMVGQGTGNPNDGYEE
                     LNLKSTKGDLQFSPWILVPHIGYGFHQYLPYPDGMSPFQAAMVDGSGYQVHRTMQFEDG
                     ASLTVNYRYTYEGSHIKGEAQVKGTGFPADGPVMTNSLTAADWCRSKKTYPNDKTIIST
                     FKWSYTTGNGKRYRSTARTTYTFAKPMAANYLKNQPMYVFRKTELKHSKTELNFKEWQK
                     AFTDVMGMDELYK"
     CDS             195..269
                     /codon_start=1
                     /label=NG1_(ohne AUG)
```

```
                     /translation="VSKGEEDNMASLPATHELHIFGSIN"
     CDS             270..350
                     /codon_start=1
                     /label=NG2
                     /translation="GVDFDMVGQGTGNPNDGYEELNLKSTK"
     CDS             351..500
                     /codon_start=1
                     /label=NG3
                     /translation="GDLQFSPWILVPHIGYGFHQYLPYPDGMSPFQAAMVDGSGYQVHR
                     TMQFE"
     CDS             501..587
                     /codon_start=1
                     /label=NG4
                     /translation="DGASLTVNYRYTYEGSHIKGEAQVKGTGF"
     CDS             588..662
                     /codon_start=1
                     /label=NG5
                     /translation="PADGPVMTNSLTAADWCRSKKTYPN"
     CDS             663..755
                     /codon_start=1
                     /label=NG6
                     /translation="DKTIISTFKWSYTTGNGKRYRSTARTTYTFA"
     CDS             756..899
                     /codon_start=1
                     /label=NG7_(ohne Stopp)
                     /translation="KPMAANYLKNQPMYVFRKTELKHSKTELNFKEWQKAFTDVMGMDE
                     LYK"
     primer_bind     complement(929..948)
                     /label=FD-Gibson-overhang rev
     primer_bind     946..968
                     /label=pGEX 3’
                     /note="pGEX vectors, reverse primer"
     primer_bind     complement(946..968)
                     /label=control on bb rev
     primer_bind     complement(1006..1024)
                     /label=pBRforEco
                     /note="pBR322 vectors, upsteam of EcoRI site, forward
                     primer"
     CDS             complement(1157..1972)
                     /codon_start=1
                     /gene="aph(3’)-Ia"
                     /product="aminoglycoside phosphotransferase"
                     /label=KanR
                     /note="confers resistance to kanamycin"
                     /translation="MSHIQRETSCSRPRLNSNMDADLYGYKWARDNVGQSGATIYRLYG
                     KPDAPELFLKHGKGSVANDVTDEMVRLNWLTEFMPLPTIKHFIRTPDDAWLLTTAIPGK
                     TAFQVLEEYPDSGENIVDALAVFLRRLHSIPVCNCPFNSDRVFRLAQAQSRMNNGLVDA
                     SDFDDERNGWPVEQVWKEMHKLLPFSPDSVVTHGDFSLDNLIFDEGKLIGCIDVGRVGI
                     ADRYQDLAILWNCLGEFSPSLQKRLFQKYGIDNPDMNKLQFHLMLDEFF"
     primer_bind     2022..2049
                     /label=front of ori fw
     primer_bind     complement(2054..2074)
                     /label=front of ori rv
     rep_origin      2100..2688
                     /direction=RIGHT
                     /label=ori
                     /note="high-copy-number ColE1/pMB1/pBR322/pUC origin of
                     replication"
     primer_bind     2589..2608
                     /label=pBR322ori-F
                     /note="pBR322 origin, forward primer"
ORIGIN
        1 gcggcctttt tacggttcct ggccttttgc tggccttttg ctcacatgtt ctttcctgcg
       61 ttatcccctg attctgtgga taaccgtatt accgccaatt gagtgagctg ataccgctcg
      121 ccgcagccga acgaccgagc gcagcgagtc acccgcgaaa tacaagaaga caattttccc
      181 gcaccagcag tggggtgtcc aagggagagg aggataatat ggcttccctg ccggcgaccc
      241 atgaacttca tatctttggt tcgatcaacg gagtggactt tgatatggtt ggtcaaggca
      301 cgggaaaccc gaacgatggg tatgaagagt tgaatcttaa atcgacaaaa ggggatcttc
```

```
      361 aattcagccc ttggattctg gtcccgcata tcgggtatgg cttccaccag tatttaccat
      421 acccggacgg gatgtctcca ttccaggccg ctatggtgga tggctctggt taccaagtgc
      481 atcgcaccat gcagttcgag gacggagcat ctcttactgt taactaccgt tatacctacg
      541 aaggatcaca cattaaggga gaagctcagg ttaaaggaac gggatttccc gctgacggac
      601 ccgtaatgac taatagcctg acagctgccg actggtgtcg cagtaagaaa acttatccta
      661 acgacaaaac gattatctcg acgttcaaat ggtcttacac gactggaaat gggaagcgct
      721 accgtagcac ggcccgcacg acgtacacct ttgccaaacc gatggcggct aactatctga
      781 agaatcaacc catgtatgtt tttcgtaaaa cagaattgaa acatagtaag actgaactga
      841 acttcaagga gtggcaaaaa gcatttacgg acgtgatggg gatggatgag ttatataaag
      901 gcattgcata aagccccgac agtaaaaaga gaccctgact agtctccggg agctgcatgt
      961 gtcagaggtt ttcaccgtca tcaccgaaac gcgcgacacg aaagggcctc gtgatacgcc
     1021 tatttttata ggttaatgtc atgataataa tggtttctta gacgtcaggt ggccttttcg
     1081 gggaaatgtg cgcggaaccc ctatttgttt atttttctaa atacattcaa atatgtatcc
     1141 gctcatgaat taattcttag aaaaactcat cgagcatcaa atgaaactgc aatttattca
     1201 tatcaggatt atcaatacca tatttttgaa aaagccgttt ctgtaatgaa ggagaaaact
     1261 caccgaggca gttccatagg atggcaagat cctggtatcg gtctgcgatt ccgactcgtc
     1321 caacatcaat acaacctatt aatttcccct cgtcaaaaat aaggttatca agtgagaaat
     1381 caccatgagt gacgactgaa tccggtgaga atggcaaaag tttatgcatt tctttccaga
     1441 cttgttcaac aggccagcca ttacgctcgt catcaaaatc actcgcatca accaaaccgt
     1501 tattcattcg tgattgcgcc tgagccagac gaaatacgcg gtcgctgtta aaaggacaat
     1561 tacaaacagg aatcgaatgc aaccggcgca ggaacaccgc cagcgcatca acaatatttt
     1621 cacctgaatc aggatattct tctaatacct ggaatgctgt tttccctggg atcgccgtgg
     1681 tgagtaacca tgcatcatca ggagtacgga taaaatgctt gatggtcgga agaggcataa
     1741 attccgtcag ccagtttagt ctgaccatct catctgtaac atcattggca acgctacctt
     1801 tgccatgttt cagaaacaac tctggcgcat cgggcttccc atacaatcga tagattgtcg
     1861 cacctgattg cccgacatta tcgcgagccc atttataccc atataaatca gcatccatgt
     1921 tggaatttaa tcgcggccta gagcaagacg tttcccgttg aatatggctc ataacacccc
     1981 ttgtattact gtttatgtaa gcagacagtt ttattgtgaa ttctcatgac caaaatccct
     2041 taacgtgagt tttcgttcca ctgagcgtca gaccccgtag aaaagatcaa aggatcttct
     2101 tgagatcctt tttttctgcg cgtaatctgc tgcttgcaaa caaaaaaacc accgctacca
     2161 gcggtggttt gtttgccgga tcaagagcta ccaactcttt ttccgaaggt aactggcttc
     2221 agcagagcgc agataccaaa tactgttctt ctagtgtagc cgtagttagg ccaccacttc
     2281 aagaactctg tagcaccgcc tacatacctc gctctgctaa tcctgttacc agtggctgct
     2341 gccagtggcg ataagtcgtg tcttaccggg ttggactcaa gacgatagtt accggataag
     2401 gcgcagcggt cgggctgaac ggggggttcg tgcacacagc ccagcttgga gcgaacgacc
     2461 tacaccgaac tgagatacct acagcgtgag ctatgagaaa gcgccacgct tcccgaaggg
     2521 agaaaggcgg acaggtatcc ggtaagcggc agggtcggaa caggagagcg cacgagggag
     2581 cttccagggg gaaacgcctg gtatctttat agtcctgtcg ggtttcgcca cctctgactt
     2641 gagcgtcgat ttttgtgatg ctcgtcaggg gggcggagcc tatggaaaaa cgccagcaac
//
```

3.0.3 p09004

```
LOCUS       Exported                3648 bp DNA     circular SYN 19-OCT-2020
DEFINITION  p09004
ACCESSION   .
VERSION     .
KEYWORDS    pNCST-mAvicFP1
SOURCE      synthetic DNA construct
  ORGANISM  synthetic DNA construct
REFERENCE   1  (bases 1 to 3648)
  TITLE     Aequorea victoria's secrets (new fluorescent proteins cloned from
            Aequorea victoria and Aequorea cf. australis)
  JOURNAL   Unpublished
FEATURES             Location/Qualifiers
     source          1..3648
                     /organism="synthetic DNA construct"
                     /mol_type="other DNA"
     primer_bind     20..39
                     /label=T7
                     /note="T7 promoter, forward primer"
     promoter        20..38
                     /label=T7 promoter
                     /note="promoter for bacteriophage T7 RNA polymerase"
     RBS             122..144
                     /note="efficient ribosome binding site from bacteriophage
                     T7 gene 10 (Olins and Rangwala, 1989)"
     CDS             164..181
```

```
                     /codon_start=1
                     /product="6xHis affinity tag"
                     /label=6xHis
                     /translation="HHHHHH"
     CDS             185..217
                     /codon_start=1
                     /product="leader peptide from bacteriophage T7 gene 10"
                     /label=T7 tag (gene 10 leader)
                     /note="promotes efficient translation in E. coli"
                     /translation="MASMTGGQQMG"
     CDS             257..277
                     /codon_start=1
                     /product="tobacco etch virus (TEV) protease recognition and
                     cleavage site"
                     /label=TEV site
                     /translation="ENLYFQG"
     CDS             278..994
                     /codon_start=1
                     /label=mAvicFP1
                     /translation="MSKGAELFNGIVPILIELNGDVHGHKFSVRGEGEGDAGSGKIEIK
                     FVCTTGTLPVPWPTLVTTLCYGVQCFTRYPEHMKQHDFYKSAMPDGYIQKRTISFQDDG
                     HYKTRAEVKFEGDTLVNRIELKGIDFKEDGNILGNKMEYNYNSHSVYVLSDKANNGIKV
                     NFKIRHNLKGEGIQLADHDQQNIPIGDGPVLLPDYHYLSTQTKITKDPNEKRDHMNLVE
                     FVTACGITHGMDELYK"
     CDS             281..346
                     /codon_start=1
                     /label=AF1_(ohne AUG)
                     /translation="SKGAELFNGIVPILIELNGDVH"
     CDS             347..427
                     /codon_start=1
                     /label=AF2
                     /translation="GHKFSVRGEGEGDAGSGKIEIKFVCTT"
     CDS             428..580
                     /codon_start=1
                     /label=AF3
                     /translation="GTLPVPWPTLVTTLCYGVQCFTRYPEHMKQHDFYKSAMPDGYIQK
                     RTISFQ"
     CDS             581..667
                     /codon_start=1
                     /label=AF4
                     /translation="DDGHYKTRAEVKFEGDTLVNRIELKGIDF"
     CDS             668..748
                     /codon_start=1
                     /label=AF5
                     /translation="KEDGNILGNKMEYNYNSHSVYVLSDKA"
     CDS             749..841
                     /codon_start=1
                     /label=AF6
                     /translation="NNGIKVNFKIRHNLKGEGIQLADHDQQNIPI"
     CDS             842..991
                     /codon_start=1
                     /label=AF7_(ohne UGA)
                     /translation="GDGPVLLPDYHYLSTQTKITKDPNEKRDHMNLVEFVTACGITHGM
                     DELYK"
     primer_bind     complement(1049..1067)
                     /label=T7 Term
                     /note="T7 terminator, reverse primer"
     terminator      1063..1110
                     /label=T7 terminator
                     /note="transcription terminator for bacteriophage T7 RNA
                     polymerase"
     rep_origin      1207..1662
                     /direction=RIGHT
                     /label=f1 ori
                     /note="f1 bacteriophage origin of replication; arrow
                     indicates direction of (+) strand synthesis"
     primer_bind     complement(1294..1313)
                     /label=F1ori-R
```

```
                     /note="F1 origin, reverse primer"
     primer_bind     1504..1525
                     /label=F1ori-F
                     /note="F1 origin, forward primer"
     promoter        1688..1792
                     /gene="bla"
                     /label=AmpR promoter
     CDS             1793..2653
                     /codon_start=1
                     /gene="bla"
                     /product="beta-lactamase"
                     /label=AmpR
                     /note="confers resistance to ampicillin, carbenicillin, and
                     related antibiotics"
                     /translation="MSIQHFRVALIPFFAAFCLPVFAHPETLVKVKDAEDQLGARVGYI
                     ELDLNSGKILESFRPEERFPMMSTFKVLLCGAVLSRIDAGQEQLGRRIHYSQNDLVEYS
                     PVTEKHLTDGMTVRELCSAAITMSDNTAANLLLTTIGGPKELTAFLHNMGDHVTRLDRW
                     EPELNEAIPNDERDTTMPVAMATTLRKLLTGELLTLASRQQLIDWMEADKVAGPLLRSA
                     LPAGWFIADKSGAGERGSRGIIAALGPDGKPSRIVVIYTTGSQATMDERNRQIAEIGAS
                     LIKHW"
     primer_bind     complement(2011..2030)
                     /label=Amp-R
                     /note="Ampicillin resistance gene, reverse primer"
     rep_origin      2824..3412
                     /direction=RIGHT
                     /label=ori
                     /note="high-copy-number ColE1/pMB1/pBR322/pUC origin of
                     replication"
     primer_bind     3313..3332
                     /label=pBR322ori-F
                     /note="pBR322 origin, forward primer"
     primer_bind     3566..3583
                     /label=L4440
                     /note="L4440 vector, forward primer"
ORIGIN
        1 gatctcgatc ccgcgaaatt aatacgactc actataggga gaccacaacg gtttccctct
       61 agaatcaccg agtttattct tgacacctga tgcgatgaat gatataatag gaaagtactg
      121 ttttgtttaa ctttaagaag gagatataca tatgcggggt tctcatcatc atcatcatca
      181 tggtatggct agcatgactg gtggacagca aatgggtcgg gatctgtacg acgatgacga
      241 taacgatccg ctcgaggaaa acctgtactt ccagggtatg tctaagggtg ctgaactgtt
      301 caacggtatc gttcctatcc tgatcgaact gaacggtgac gttcatggtc ataagttctc
      361 tgttcgtggt gaaggtgaag gtgacgctgg ttctggtaag atcgaaatca agttcgtttg
      421 caccaccggt accctgcctg ttccttggcc taccctggtt accaccttat gctacggtgt
      481 tcagtgcttc acccgttacc ctgaacatat gaagcagcat gacttctaca agtctgctat
      541 gcctgacggt tacatccaga agcgtaccat ctctttccag gacgacggtc attacaagac
      601 ccgtgctgaa gttaagttcg aaggtgacac cctggttaac cgtatcgaac tgaagggtat
      661 cgacttcaag gaagacggta acatcctggg taacaagatg gaatacaact acaactctca
      721 ttctgtttac gttctgtctg acaaggctaa caacggtatc aaggttaact tcaagatccg
      781 tcataacctg aagggtgaag gtatccagct ggctgaccat gaccagcaga acatccctat
      841 cggtgacggt cctgttctgc tgcctgacta ccattacctg tctacccaga ccaagatcac
      901 caaggaccct aacgaaaagc gtgaccatat gaacctggtt gaattcgtta ccgcttgcgg
      961 tatcacccat ggtatggacg aactgtacaa gtgacgtttg atccggctgc taacaaagcc
     1021 cgaaaggaag ctgagttggc tgctgccacc gctgagcaat aactagcata accccttggg
     1081 gcctctaaac gggtcttgag gggttttttg ctgaaaggag gaactatatc cggatctggc
     1141 gtaatagcga agaggcccgc accgatcgcc cttcccaaca gttgcgcagc ctgaatggcg
     1201 aatgggacgc gccctgtagc ggcgcattaa gcgcggcggg tgtggtggtt acgcgcagcg
     1261 tgaccgctac acttgccagc gccctagcgc ccgctccttt cgctttcttc ccttcctttc
     1321 tcgccacgtt cgccggcttt ccccgtcaag ctctaaatcg ggggctccct ttagggttcc
     1381 gatttagtgc tttacggcac ctcgacccca aaaaacttga ttagggtgat ggttcacgta
     1441 gtgggccatc gccctgatag acggtttttc gccctttgac gttggagtcc acgttcttta
     1501 atagtggact cttgttccaa actggaacaa cactcaaccc tatctcggtc tattcttttg
     1561 atttataagg gattttgccg atttcggcct attggttaaa aaatgagctg atttaacaaa
     1621 aatttaacgc gaattttaac aaaatattaa cgcttacaat ttaggtggca cttttcgggg
     1681 aaatgtgcgc ggaaccccta tttgtttatt tttctaaata cattcaaata tgtatccgct
     1741 catgagacaa taaccctgat aaatgcttca ataatattga aaaaggaaga gtatgagtat
     1801 tcaacatttc cgtgtcgccc ttattccctt ttttgcggca ttttgccttc ctgtttttgc
     1861 tcacccagaa acgctggtga aagtaaaaga tgctgaagat cagttgggtg cacgagtggg
     1921 ttacatcgaa ctggatctca acagcggtaa gatccttgag agttttcgcc ccgaagaacg
```

```
     1981 ttttccaatg atgagcactt ttaaagttct gctatgtggc gcggtattat cccgtattga
     2041 cgccgggcaa gagcaactcg gtcgccgcat acactattct cagaatgact tggttgagta
     2101 ctcaccagtc acagaaaagc atcttacgga tggcatgaca gtaagagaat tatgcagtgc
     2161 tgccataacc atgagtgata acactgcggc caacttactt ctgacaacga tcggaggacc
     2221 gaaggagcta accgcttttt tgcacaacat gggggatcat gtaactcgcc ttgatcgttg
     2281 ggaaccggag ctgaatgaag ccataccaaa cgacgagcgt gacaccacga tgcctgtagc
     2341 aatggcaaca acgttgcgca aactattaac tggcgaacta cttactctag cttcccggca
     2401 acaattaata gactggatgg aggcggataa agttgcagga ccacttctgc gctcggccct
     2461 tccggctggc tggtttattg ctgataaatc tggagccggt gagcgtgggt ctcgcggtat
     2521 cattgcagca ctggggccag atggtaagcc ctcccgtatc gtagttatct acacgacggg
     2581 gagtcaggca actatggatg aacgaaatag acagatcgct gagataggtg cctcactgat
     2641 taagcattgg taactgtcag accaagttta ctcatatata ctttagattg atttaaaact
     2701 tcatttttaa tttaaaagga tctaggtgaa gatccttttt gataatctca tgaccaaaat
     2761 cccttaacgt gagttttcgt tccactgagc gtcagacccc gtagaaaaga tcaaaggatc
     2821 ttcttgagat cctttttttc tgcgcgtaat ctgctgcttg caaacaaaaa aaccaccgct
     2881 accagcggtg gtttgtttgc cggatcaaga gctaccaact ctttttccga aggtaactgg
     2941 cttcagcaga gcgcagatac caaatactgt tcttctagtg tagccgtagt taggccacca
     3001 cttcaagaac tctgtagcac cgcctacata cctcgctctg ctaatcctgt taccagtggc
     3061 tgctgccagt ggcgataagt cgtgtcttac cgggttggac tcaagacgat agttaccgga
     3121 taaggcgcag cggtcggact gaacgggggg ttcgtgcaca cagcccagct tggagcgaac
     3181 gacctacacc gaactgagat acctacagcg tgagctatga gaaagcgcca cgcttcccga
     3241 agggagaaag gcggacaggt atccggtaag cggcagggtc ggaacaggag agcgcacgag
     3301 ggagcttcca gggggaaacg cctggtatct ttatagtcct gtcgggtttc gccacctctg
     3361 acttgagcgt cgatttttgt gatgctcgtc aggggggcgg agcctatgga aaaacgccag
     3421 caacgcggcc tttttacggt tcctggcctt ttgctggcct tttgctcaca tgttctttcc
     3481 tgcgttatcc cctgattctg tggataaccg tattaccgcc tttgagtgag ctgataccgc
     3541 tcgccgcagc cgaacgaccg agcgcagcga gtcagtgagc gaggaagcgg aagagcgccc
     3601 aatacgcaaa ccgcctctcc ccgcgcgttg gccgattcat taatgcag
//
```

3.0.4 p09005

```
LOCUS       Exported                3740 bp DNA     circular SYN 11-JAN-2021
DEFINITION  p09005
ACCESSION   .
VERSION     .
KEYWORDS    .
SOURCE      synthetic DNA construct
  ORGANISM  synthetic DNA construct
REFERENCE   1  (bases 1 to 3740)
FEATURES             Location/Qualifiers
     source          1..3740
                     /organism="synthetic DNA construct"
                     /mol_type="other DNA"
     source          797..839
                     /label=synthetic DNA construct
     source          840..1538
                     /label=synthetic DNA construct
     misc_feature    1..35
                     /label=Promoter-J23119(SpeI)
     primer_bind     4..57
                     /label=BO_09191_J23119
     primer_bind     complement(6..25)
                     /label=09190_J23119_rev
     primer_bind     8..25
                     /label=09192_CPCR_fw
     primer_bind     26..46
                     /label=09189_J23119_fw
     misc_feature    32..35
                     /label=BbsI
     primer_bind     complement(61..82)
                     /label=09006_p09001-Vektor_rev
     RBS             64..71
                     /label=Untitled annotation
     CDS             80..796
                     /codon_start=1
                     /label=sfGFP
                     /translation="MSKGEELFTGVVPILVELDGDVNGHKFSVRGEGEGDATNGKLTLK
```

```
                     FICTTGKLPVPWPTLVTTLTYGVQCFSRYPDHMKRHDFFKSAMPEGYVQERTISFKDDG
                     TYKTRAEVKFEGDTLVNRIELKGIDFKEDGNILGHKLEYNFNSHNVYITADKQKNGIKA
                     NFKIRHNVEDGSVQLADHYQQNTPIGDGPVLLPDNHYLSTQSVLSKDPNEKRDHMVLLE
                     FVTAAGITHGMDELYK"
     CDS             83..148
                     /codon_start=1
                     /label=GFP1_(ohne AUG)
                     /translation="SKGEELFTGVVPILVELDGDVN"
     CDS             149..229
                     /codon_start=1
                     /label=GFP2
                     /translation="GHKFSVRGEGEGDATNGKLTLKFICTT"
     CDS             230..382
                     /codon_start=1
                     /label=GFP3
                     /translation="GKLPVPWPTLVTTLTYGVQCFSRYPDHMKRHDFFKSAMPEGYVQE
                     RTISFK"
     CDS             383..469
                     /codon_start=1
                     /label=GFP4
                     /translation="DDGTYKTRAEVKFEGDTLVNRIELKGIDF"
     misc_feature    complement(397..414)
                     /label=10296_sGFP_rv
     primer_bind     415..437
                     /label=10295_sGFP_fw
     CDS             470..550
                     /codon_start=1
                     /label=GFP5
                     /translation="KEDGNILGHKLEYNFNSHNVYITADKQ"
     CDS             551..643
                     /codon_start=1
                     /label=GFP6
                     /translation="KNGIKANFKIRHNVEDGSVQLADHYQQNTPI"
     CDS             644..793
                     /codon_start=1
                     /label=GFP7_(ohne UAA)
                     /translation="GDGPVLLPDNHYLSTQSVLSKDPNEKRDHMVLLEFVTAAGITHGM
                     DELYK"
     primer_bind     771..836
                     /label=BO_09068_Vektor-5UTR
     primer_bind     complement(774..796)
                     /label=09004_p09001-Vektor_rev
     primer_bind     794..820
                     /label=09005_p09001-Vektor_fw
     misc_feature    797..1538
                     /label=Geneious type: Editing History Insertion
     5'UTR           797..839
                     /label=5'-UTR
     RBS             811..833
                     /label=efficient ribosome binding site from bacterioph
                     /label=efficient ribosome binding site from bacteriophageT7
                     gene 10 (Olins and Rangwala, 1989)
     gene            840..1538
                     /label=mKate2
     primer_bind     840..860
                     /label=11316
     primer_bind     1444..1462
                     /label=11319
     primer_bind     1519..1561
                     /label=BO_09070_mKate2-Vektor
     primer_bind     complement(1520..1538)
                     /label=11317
     primer_bind     complement(1525..1538)
                     /label=09163_mKate-ORF_rev
     primer_bind     1539..1559
                     /label=09003_p09001-Vektor_fw
     3'UTR           1539..1552
                     /label=3'-UTR
```

```
     misc_feature    complement(1547..1552)
                     /label=BamHI-BglII Scar(4)
     misc_feature    1547..1550
                     /label=BbsI
     misc_feature    1553..1582
                     /label=t500
     primer_bind     1579..1623
                     /label=10448_LCR_tGFP-BB1_fw
     misc_feature    complement(1583..1604)
                     /label=10442_tGFP_rv
     primer_bind     1605..1619
                     /label=10443_BB1_fw
     rep_origin      1768..2450
                     /label=Untitled annotation
     misc_feature    complement(2366..2371)
                     /label=BamHI-BglII Scar(5)
     misc_feature    2377..2382
                     /label=BamHI-BglII Scar(1)
     misc_feature    complement(2463..2468)
                     /label=BamHI-BglII Scar(6)
     misc_feature    2475..2480
                     /label=BamHI-BglII Scar(2)
     CDS             complement(2711..3207)
                     /codon_start=1
                     /label=AmpR
                     /translation="MSTFKVLLCGAVLSRIDAGQEQLGRRIHYSQNDLVEYSPVTEKHL
                     TDGMTVRELCSAAITMSDNTAANLLLTTIGGPKELTAFLHNMGDHVTRLDRWEPELNEA
                     IPNDERDTTMPVAMATTLRKLLTGELLTLASRQQLIDWMEADKVAGPLLRSALPAGWFI
                     AD"
     primer_bind     2924..2971
                     /label=10449_LCR_BB1-BB2_fw
     misc_feature    complement(2929..2946)
                     /label=10444_BB1_rv
     primer_bind     2947..2965
                     /label=10445_BB2_fw
     misc_feature    complement(3243..3248)
                     /label=BamHI-BglII Scar(7)
     misc_feature    complement(3243..3248)
                     /label=BamHI-BglII Scar
     misc_feature    3260..3265
                     /label=BamHI-BglII Scar(3)
     misc_feature    complement(3623..3644)
                     /label=10446_BB2_rv
     primer_bind     3629..3684
                     /label=2020-11-24_BO-1039
     primer_bind     3629..3684
                     /label=09171_BO_Vek-T7
     primer_bind     3661..3671
                     /label=09161_T7_fw
     primer_bind     3691..3710
                     /label=T7
     promoter        3691..3709
                     /label=T7 promoter
     primer_bind     join(3717..3740,1..30)
                     /label=2020-11-24_BO-1040
     primer_bind     join(3717..3740,1..30)
                     /label=09172_BO_T7-SpeI
     primer_bind     complement(3722..3740)
                     /label=09162_T7_rev
ORIGIN
        1 ttgacagcta gctcagtcct aggtataata ctagtatgtc ttcggatctt agctactaga
       61 gaaagaggag aaatactaga tgagcaaagg agaagaactt ttcactggag ttgtcccaat
      121 tcttgttgaa ttagatggtg atgttaatgg gcacaaattt tctgtccgtg gagagggtga
      181 aggtgatgct acaaacggaa aactcaccct taaatttatt tgcactactg gaaaactacc
      241 tgttccgtgg ccaacacttg tcactactct gacctatggt gttcaatgct tttcccgtta
      301 tccggatcac atgaaacggc atgacttttt caagagtgcc atgcccgaag gttatgtaca
      361 ggaacgcact atatctttca aagatgacgg gacctacaag acgcgtgctg aagtcaagtt
      421 tgaaggtgat acccttgtta atcgtatcga gttaaagggt attgatttta aagaagatgg
```

```
     481 aaacattctt ggacacaaac tcgagtacaa ctttaactca cacaatgtat acatcacggc
     541 agacaaacaa aagaatggaa tcaaagctaa cttcaaaatt cgccacaacg ttgaagatgg
     601 ttccgttcaa ctagcagacc attatcaaca aaatactcca attggcgatg gccctgtcct
     661 tttaccagac aaccattacc tgtcgacaca atctgtcctt tcgaaagatc ccaacgaaaa
     721 gcgtgaccac atggtccttc ttgagtttgt aactgctgct gggattacac atggcatgga
     781 tgagctctac aaataacctc tagaaataat tttgtttaac tttaagaagg agatatacca
     841 tggtgagcga gctgattaag gagaacatgc acatgaagct gtacatggag ggcaccgtga
     901 acaaccacca cttcaagtgc acatccgagg gcgaaggcaa gccctacgag ggcacccaga
     961 ccatgagaat caaggccgtc gagggcggcc ctctcccctt cgccttcgac atcctggcta
    1021 ccagcttcat gtacggcagc aaaaccttca tcaaccacac ccagggcatc cccgacttct
    1081 ttaagcagtc cttccctgag ggcttcacat gggagagagt caccacatac gaagacgggg
    1141 gcgtgctgac cgctacccag gacaccagcc tccaggacgg ctgcctcatc tacaacgtca
    1201 agatcagagg ggtgaacttc ccatccaacg gccctgtgat gcagaagaaa acactcggct
    1261 gggaggcctc caccgagacc ctgtaccccg ctgacggcgg cctggaaggc agagccgaca
    1321 tggccctgaa gctcgtgggc gggggccacc tgatctgcaa cttgaagacc acatacagat
    1381 ccaagaaacc cgctaagaac ctcaagatgc ccggcgtcta ctatgtggac agaagactgg
    1441 aaagaatcaa ggaggccgac aaagagacct acgtcgagca gcacgaggtg gctgtggcca
    1501 gatactgcga cctccctagc aaactggggc acagatgaga agacaaggat ctcaaagccc
    1561 gccgaaaggc gggctttttt ttggatcctt actcgagtct agactgcagg cttcctcgct
    1621 cactgactcg ctgcgctcgg tcgttcggct gcggcgagcg gtatcagctc actcaaaggc
    1681 ggtaatacgg ttatccacag aatcagggga taacgcagga aagaacatgt gagcaaaagg
    1741 ccagcaaaag gccaggaacc gtaaaaaggc cgcgttgctg gcgtttttcc acaggctccg
    1801 cccccctgac gagcatcaca aaaatcgacg ctcaagtcag aggtggcgaa acccgacagg
    1861 actataaaga taccaggcgt ttccccctgg aagctccctc gtgcgctctc ctgttccgac
    1921 cctgccgctt accggatacc tgtccgcctt tctcccttcg ggaagcgtgg cgctttctca
    1981 tagctcacgc tgtaggtatc tcagttcggt gtaggtcgtt cgctccaagc tgggctgtgt
    2041 gcacgaaccc cccgttcagc ccgaccgctg cgccttatcc ggtaactatc gtcttgagtc
    2101 caacccggta agacacgact tatcgccact ggcagcagcc actggtaaca ggattagcag
    2161 agcgaggtat gtaggcggtg ctacagagtt cttgaagtgg tggcctaact acggctacac
    2221 tagaagaaca gtatttggta tctgcgctct gctgaagcca gttaccttcg gaaaaagagt
    2281 tggtagctct tgatccggca aacaaaccac cgctggtagc ggtggttttt ttgtttgcaa
    2341 gcagcagatt acgcgcagaa aaaaaggatc tcaagaagat cctttgatct tttctacggg
    2401 gtctgacgct cagtggaacg aaaactcacg ttaagggatt ttggtcatga gattatcaaa
    2461 aaggatcttc acctagatcc ttttaaatta aaaatgaagt tttaaatcaa tctaaagtat
    2521 atatgagtaa acttggtctg acagttacca atgcttaatc agtgaggcac ctatctcagc
    2581 gatctgtcta tttcgttcat ccatagttgc ctgactcccc gtcgtgtaga taactacgat
    2641 acgggagggc ttaccatctg gccccagtgc tgcaatgata ccgcgagacc cacgctcacc
    2701 ggctccagat ttatcagcaa taaaccagcc agccggaagg gccgagcgca gaagtggtcc
    2761 tgcaacttta tccgcctcca tccagtctat taattgttgc cgggaagcta gagtaagtag
    2821 ttcgccagtt aatagtttgc gcaacgttgt tgccattgct acaggcatcg tggtgtcacg
    2881 ctcgtcgttt ggtatggctt cattcagctc cggttcccaa cgatcaaggc gagttacatg
    2941 atcccccatg ttgtgcaaaa aagcggttag ctccttcggt cctccgatcg ttgtcagaag
    3001 taagttggcc gcagtgttat cactcatggt tatggcagca ctgcataatt ctcttactgt
    3061 catgccatcc gtaagatgct tttctgtgac tggtgagtac tcaaccaagt cattctgaga
    3121 atagtgtatg cggcgaccga gttgctcttg cccggcgtca atacgggata ataccgcgcc
    3181 acatagcaga actttaaaag tgctcatcat tggaaaacgt tcttcggggc gaaaactctc
    3241 aaggatctta ccgctgttga gatccagttc gatgtaaccc actcgtgcac ccaactgatc
    3301 ttcagcatct tttactttca ccagcgtttc tgggtgagca aaaacaggaa ggcaaaatgc
    3361 cgcaaaaaag ggaataaggg cgacacggaa atgttgaata ctcatactct tcctttttca
    3421 atattattga agcatttatc agggttattg tctcatgagc ggatacatat ttgaatgtat
    3481 ttagaaaaat aaacaaatag gggttccgcg cacatttccc cgaaaagtgc cacctgacgt
    3541 ctaagaaacc attattatca tgacattaac ctataaaaat aggcgtatca cgaggcagaa
    3601 tttcagataa aaaaaatcct tagctttcgc taaggatgat ttctggaatt ctaaagatct
    3661 cattaatgca ggatctcgat cccgcgaaat taatacgact cactataggg agaccacaac
    3721 ggtttccctc tagaatcacc
//
```

4 Comparisons and Costs

4.1 Method Comparison

Comparison of different RCA-CFPS systems

Tab. 1: Comparison of workflow steps between HyperXpress and other RCA-CFPS systems
*Assumption: The over night incubation represents a period of 12 h.
**The RCA was carried out in a 100 µl reaction whereas the CFPS took place in a 50 µl reaction. This means that only an aliquot of the 100 µl-RCA can be used for the 50 µl-CFPS.
(t: Time required for a workflow step)

**Workflow step**	**HyperXpress**	**RCA-CFPS system of ref. [1,2]**	**RCA-CFPS system of ref. [3]**
**0**	Preparations<br>(PCR amplification of DNA fragments)	Preparations<br>(ordering of DNA expression construct)	Preparations<br>(ordering of DNA expression construct)
**1**	LCR<br>(t: 0.75 h)	PCR amplification of DNA construct<br>(t: 1.6 h)	BamHI and BglII digestion of DNA construct<br>(t: Not specified)
**2**	RCA<br>(t: 3.25 h)	Purification of the PCR product<br>(t: Not specified)	T4 ligation for circularization<br>(t: 1.8 h)
**3**	PEG DNA precipitation (optional)<br>(t: 1 h)	HindIII digestion of amplificate's ends<br>(t: 1.3 h)	Exonuclease I and exonuclease III digestion of linear DNA (byproducts)<br>(t: 1.3 h)
**4**	CFPS<br>(t: 5 h)	T4 ligation for circularization<br>(t: 1 h)	RCA<br>(t: over night ≈ 12 h*)
**5**	/	Purification and dilution of circular DNA<br>(t: Not specified)	Determining the RCA-DNA product concentration and transfering the RCA volume for 0.5 µg RCA-DNA in another vessel for the CFPS**<br>(t: Not specified)
**6.**	/	RCA<br>(t: over night ≈ 12 h* or in 4-6 h on the same day)	CFPS (followed by an assay)<br>(t: 6 h without the assay)
**7.**	/	Purification of the RCA product<br>(t: Not specified)	/
**8.**	/	CFPS<br>(t: 4 h)	/

Tab. 2: Comparison of workflow steps between HyperXpress and other RCA-CFPS systems
*The number of steps means all steps of the workflow in Tab. 1 without step 0.
**For the ref. [1,2], the minimal possible RCA time of 4 h is used for the calculation of t_{total}.
(t_{total}: Time required for the whole workflow without step 0; steps without specified time are not integrated)

	**HyperXpress**	**RCA-CFPS system of ref. [1,2]**	**RCA-CFPS system of ref. [3]**
**Number of steps***	4	8	6
**t_{total} in h**	10	11.9**	21.1

References:
[1]: Dopp JL, Rothstein SM, Mansell TJ, and Reuel NF. Rapid prototyping of proteins: Mail order gene fragments to assayable proteins within 24 hours. Biotechnology and Bioengineering. 2019;116:667–676. https://doi.org/10.1002/bit.26912
[2]: Dopp JL, Jo YR, and Reuel NF. Methods to reduce variability in E. Coli-based cell-free protein expression experiments. Synth Syst Biotechnol. 2019 Dec; 4(4): 204–211. DOI: 10.1016/j.synbio.2019.10.003
[3]: Hadi, T., Nozzi, N., Melby, J.O. et al. Rolling circle amplification of synthetic DNA accelerates biocatalytic determination of enzyme activity relative to conventional methods. Sci Rep 10, 10279 (2020). https://doi.org/10.1038/s41598-020-67307-9

4.2 Costs Calculations

Cost calculation for HyperXpress

The whole cost calculation is only based on the reagent consumption and not on the working time because costs associated with the working time depend on the working speed and the hourly wages which are highly individual parameters.

Note: The costs for the used MilliQ H_2O are not included because of a too low price of the in house-made water.

Abbreviations:
c_{final}: Final concentration of the component in the reaction
V: Reaction volume
Z: Amount of the component in the volume V (e.g. in g, mol, U depending on the component)
P: Price per individual unit of the component (e.g. in €/g, €/mol, €/l depending on the component)
(P is calculated as the quotient of the component's price in € and the amount of the component e.g. in mol)
prec.: PEG DNA precipitation
K: Costs of the component in the reaction in €
ON: Ordering number
n: Amount of substance in mol
m: Mass in g
M: Molar mass in g/mol

0. Costs for the DNA fragments

0.1. Costs for the primer 5'-phosphorylation

Tab. 1: List of all components required for the 5'-phosphorylation of primers
*Assumption: All PCR primers have an average length of 24 nt with a GC content of 50%.

**Component**	**Price from 12/27/21 in €**	**Price per unit of the individual component (P)**
DNA primer (Sigma-Aldrich, 0.025 µmol, desalted, dry in tubes)*	9.84	$3.936 \cdot 10^8$ €/mol
T4 Polynucleotide Kinase / T4-PNK (NEB, ON: M0201S) (10 U/µl, 500 U in 50 µl)	58	0.116 €/U
10x T4 Polynucleotide Kinase Reaction Buffer / T4-PNK buffer (NEB) (10x T4-PNK buffer is delivered together with T4-PNK in one kit)	/	/
ATP disodium salt (Carl Roth, ON: HN35.1) (5 g ATP => n=m/M=5 g/551.10 g/mol=$9.07 \cdot 10^{-3}$ mol ATP)	17.90	1974 €/mol

Tab. 2: Cost calculations for the 5'-phosphorylation of primers (V=50 µl per reaction)

**Component**	**c_{final} in the phosphorylation reaction**	**$Z = c_{final} \cdot V$ (V=50 µl=$5 \cdot 10^{-5}$ l)**	**P (see Tab. 1)**	**$K = Z \cdot P = c_{final} \cdot V \cdot P$ in €**
T4-PNK	$2 \cdot 10^5$ U/l (=0.2 U/µl)	10 U	0.116 €/U	1.16
T4-PNK buffer	1x	/	/	/
ATP	$1.7 \cdot 10^{-3}$ mol/l (=1.7 mM)	$8.5 \cdot 10^{-8}$ mol	1974 €/mol	0.0002
DNA primer	10^{-5} mol/l (=10 µM)	$5 \cdot 10^{-10}$ mol	$3.936 \cdot 10^8$ €/mol	0.197
MilliQ H_2O	/	Not included in the calculation	Not included in the calculation	Not included in the calculation
**Sum**:	/	/	/	**1.357**
**P(5'-phosphorylated primer)=K/Z(primer)=1.357 €/($5 \cdot 10^{-10}$ mol)=$2.714 \cdot 10^9$ €/mol≈0.027 €/nmol**				

<u>Costs of primer 5'-phosphorylation</u>: **1.357 € per 50 µl reaction** => **0.027 €/nmol 5'-phosphorylated / 5'P-primer**

0.2. Costs for the PCR amplification of the DNA fragments

Tab. 3: List of all components required for the PCR amplification of DNA fragments

**Component**	**Price from 12/27/21 in €**	**Price per unit of the individual component (P)**
5'P-primer (see Tab. 2)	/	$2.714 \cdot 10^{9}$ €/mol
Q5® High-Fidelity DNA Polymerase / Q5® DNAP (NEB, ON: M0491L) (2 U/µl, 500 U)	435	0.87 €/U
Q5® Reaction Buffer Pack / Q5® buffer (NEB) (5x Q5® buffer is delivered together with Q5® DNAP in one kit)	/	/
dNTP-Set 1 (Carl Roth, ON: K039.2) (5x 4 dNTPs with 25 µmol each)	419	$1.676 \cdot 10^{7}$ €/mol

Tab. 4: Cost calculations for the PCR amplification of DNA fragments (V=50 µl per reaction)
fw-primer: forward primer; rev-primer: reverse primer
*The concentration of the DNA template is far too low to have a relevant impact on the costs.

**Component**	**c_{final} in the PCR**	**$Z = c_{final} \cdot V$ (V=50 µl=$5 \cdot 10^{-5}$ l)**	**P (see Tab. 3)**	**$K = Z \cdot P = c_{final} \cdot V \cdot P$ in €**
Q5® DNAP	$4 \cdot 10^{4}$ U/l (=0.04 U/µl)	2 U	0.87 €/U	1.74
Q5® buffer	1x	/	/	/
dNTP	$2 \cdot 10^{-4}$ mol/l each (=0.2 mM each)	10^{-8} mol	$1.676 \cdot 10^{7}$ €/mol	0.168
DNA template*	10^{-15} mol/l (=1 fM)	Not included in the calculation	Not included in the calculation	Not included in the calculation
5'P-fw primer	$25 \cdot 10^{-8}$ mol/l (=250 nM)	$1.25 \cdot 10^{-11}$ mol	$2.714 \cdot 10^{9}$ €/mol	0.034
5'P-rev primer	$25 \cdot 10^{-8}$ mol/l (=250 nM)	$1.25 \cdot 10^{-11}$ mol	$2.714 \cdot 10^{9}$ €/mol	0.034
MilliQ H_2O	/	Not included in the calculation	Not included in the calculation	Not included in the calculation
**Sum**:	/	/	/	**1.976**

<u>**Costs of PCR amplification**</u>: **1.976 € per 50 µl reaction** => **K/V=1.976 €/50 µl=0.040 €/µl for every 50 µl PCR**

0.3. Costs for the DpnI digestion of the unpurified PCR product

Tab. 5: List of all components required for the DpnI digestion

Component	Price from 12/27/21 in €	Price per unit of the individual component (P)
Unpurified PCR product<br>(see "Costs of PCR amplification" under Tab. 4)	/	0.04 €/µl
DpnI (NEB, ON: R0176L) (20 U/µl, 5000 U)	264	0.053 €/U
CutSmart™ Buffer (NEB)<br>(10x CutSmart™ Buffer is delivered together with DpnI in one kit)	/	/

Tab. 6: Cost calculations for the DpnI digestion of the unpurified PCR product (V=55 µl per DpnI digestion)
*Only 47 µl of the PCR is used for the DpnI digestion because the other 3 µl of the 50 µl are necessary for verifying the PCR product via an agarose gel electrophoresis. Despite this, the calculation is carried out with a 50 µl PCR to include the costs for the whole PCR including the amount necessary for gel verification.

Component	c_{final} in the digestion reaction	$Z = c_{final} \cdot V$ ($V=55\ \mu l=5.5\cdot10^{-5}\ l$)	P (see Tab. 5)	$K = Z\cdot P = c_{final}\cdot V\cdot P$ in €
PCR product*	/ (50 µl*)	/	0.04 €/µl*	1.976*<br>(for the 50 µl PCR)
DpnI	$4\cdot10^5$ U/l<br>(=0.4 U/µl)	22 U	0.053 €/U	1.166
CutSmart™ Buffer	1x	/	/	/
MilliQ H_2O	/	Not included in the calculation	Not included in the calculation	Not included in the calculation
**Sum**:	/	/	/	**3.142**

<u>Costs of DpnI digestion</u>: **3.142 € per 55 µl reaction** => **K/V=3.142 €/55 µl=0.057 €/µl for every 55 µl digestion**

0.4. Costs for the purification of the DpnI digested, unpurified PCR product

Tab. 7: List of all components required for the PCR purification

Component	Price from 12/27/21 in €	Price per unit of the individual component (P)
innuPREP PCRpure Kit (Analytik Jena GmbH, ON: 845-KS-5010250) (250 columns) (source of price: neoLab®, article number: C-6114)	343.91	1.376 €/column
DpnI digested, unpurified PCR product (see "Costs of DpnI digestion" under Tab. 6)	/	0.057 €/µl

Tab. 8: Cost calculations for the purification of two DpnI digested PCR products via one column*
*Two DpnI digested PCR products (2x 55 µl = 110 µl) are purified via one column to increase the concentration of the eluted, purified DNA fragment.

Component	c_{final} in the purification	$Z = c_{final} \cdot V$	P (see Tab. 7)	$K = Z \cdot P = c_{final} \cdot V \cdot P$ in €
DpnI digested PCR product*	/ (110 µl*)	/	0.057 €/µl*	6.284* (for 110 µl)
PCRpure Kit	/ (1 column)	/	1.376 €/column	1.376
**Sum**:	/	/	/	**7.66**

Costs of DNA fragment purification (V_{eluate}=25 µl):
7.66 € per 25 µl DNA eluate => **K/V=7.66 €/25 µl=0.306 €/µl for every 25 µl DNA eluate**

Costs of vector purification:
(V_{eluate}=25 µl, vector concentration in the eluate c_{eluate}=25 nM, vector length l=3029 bp)
($n_{vector}=c_{eluate} \cdot V_{eluate}=25 \cdot 10^{-9}$ mol/l $\cdot$ $25 \cdot 10^{-6}$ l=$0.625 \cdot 10^{-12}$ mol=0.625 pmol)
K/n_{vector}=7.66 €/($0.625 \cdot 10^{-12}$ mol)=$12.256 \cdot 10^{12}$ €/mol=12.256 €/pmol for every 25 µl vector eluate

Costs of insert purification:
(V_{eluate}=25 µl, insert concentration in the eluate c_{eluate}=300 nM, average insert length l=102 bp)
($n_{insert}=c_{eluate} \cdot V_{eluate}=300 \cdot 10^{-9}$ mol/l $\cdot$ $25 \cdot 10^{-6}$ l=$7.5 \cdot 10^{-12}$ mol=7.5 pmol)
K/n_{insert}=7.66 €/($7.5 \cdot 10^{-12}$ mol)=$1.021 \cdot 10^{12}$ €/mol=1.021 €/pmol for every 25 µl insert eluate

1. Costs for the 0.612 µl LCR

Tab. 9: List of all components required for the LCR

*Assumption: All BOs have an average length of 50 nt with a GC content of 50%.

**Only the price in $ was available for the Ampligase®, which is why it is converted in € through the current exchange rate of 1 $ ~ 0.88 €[1] from 12/28/21.

***For better comparability, the official price of the commercially available buffer is used to calculate costs.

**Component**	**Price from 12/28/21 in €**	**Price per unit of the individual component (P)**
vector DNA fragment<br>(see "Costs of vector purification" under Tab. 8)	/	$12.256 \cdot 10^{12}$ €/mol<br>(=12.256 €/pmol)
insert DNA fragment<br>(see "Costs of insert purification" under Tab. 8)	/	$1.021 \cdot 10^{12}$ €/mol<br>(=1.021 €/pmol)
BO (Sigma-Aldrich, 0.025 µmol, desalted, dry in tubes)*	20.50	$0.82 \cdot 10^{9}$ €/mol<br>(=0.82 €/nmol)
Ampligase® Thermostable DNA Ligase (Lucigen, ON: A3210K)<br>(5 U/µl, 10000 U)	624.8<br>(~ 710 $)**	0.062 €/U
10x Ampligase® buffer (self-made)***<br>(5 ml 10x Ampligase® buffer for 68 $, Lucigen, ON: A1905B)	59.84<br>(~ 68 $)	11968 €/l
$MgCl_2 \cdot 6\,H_2O$ (Carl Roth, ON: 2189.1)<br>(1 kg; n=m/M=1000 g/203.3 g/mol=4.919 mol)	30.90	6.282 €/mol
NAD^+ (Sigma-Aldrich, ON: N7004-1g)<br>(1 g; n=m/M=1 g/663.43 g/mol=$1.507 \cdot 10^{-3}$ mol)	67.90	$45.056 \cdot 10^{3}$ €/mol

Tab. 10: Cost calculations for the LCR (V=0.612 µl)
*The calculations are carried out for the assembly of 8 DNA fragments (1 vector, 7 inserts) through 8 BOs to form a circular product.

**Component**	**c_{final} in the LCR**	**$Z = c_{final} \cdot V$ ($V=0.612 \cdot 10^{-6}$ l)**	**P (see Tab. 9)**	**$K = Z \cdot P = c_{final} \cdot V \cdot P$ in €**
DNA fragments*				
1 vector	$3 \cdot 10^{-9}$ mol/l (=3 nM)	$1.836 \cdot 10^{-15}$ mol	$12.256 \cdot 10^{12}$ €/mol	0.023
7 inserts	$3 \cdot 10^{-9}$ mol/l each (=3 nM each)	$1.836 \cdot 10^{-15}$ mol each => $12.852 \cdot 10^{-15}$ mol for 7 inserts	$1.021 \cdot 10^{12}$ €/mol	0.013 for 7 inserts
BO (8 BOs)	$3 \cdot 10^{-8}$ mol/l (=30 nM each)	$1.836 \cdot 10^{-14}$ mol each => $14.688 \cdot 10^{-14}$ mol for 8 BO	$0.82 \cdot 10^{9}$ €/mol	0.0001 for 8 BOs
Ampligase® buffer	1x	/ ($0.612 \cdot 10^{-7}$ l 10x buffer)	11968 €/l	0.0007
$MgCl_2$	0.01 mol/l (=10 mM)	$0.612 \cdot 10^{-8}$ mol	6.282 €/mol	<0.0001
NAD^+	$5 \cdot 10^{-4}$ mol/l (=0.5 mM)	$3.06 \cdot 10^{-10}$ mol	$45.056 \cdot 10^{3}$ €/mol	<0.0001
Ampligase®	$3 \cdot 10^{5}$ U/l (=0.3 U/µl)	0.1836 U	0.062 €/U	0.011
MilliQ H_2O	/	Not included in the calculation	Not included in the calculation	Not included in the calculation
**Sum**:	/	/	/	**0.048**

<u>Costs of LCR</u>: **0.048 € per 0.612 µl LCR** => **K/V=0.048 €/0.612 µl=0.078 €/µl for every 0.612 µl LCR**

2. Costs for the 1.8 µl RCA

Tab. 11: List of all components required for the RCA
*Assumption: The price of the phi29 buffer corresponds to a third of the price for the phi29-DNAP from NEB
(60 € for 250 U phi29 DNAP + phi29 buffer + 600 µl BSA => 20 € for the phi29 buffer).
Reason for the assumption: There is no single price for the phi29 buffer available.

**Component**	**Price from 12/28/21 in €**	**Price per unit of the individual component (P)**
phi29 DNA Polymerase Reaction Buffer / phi29 buffer (NEB, ON: B0269SVIAL) (1.5 ml=$1.5\cdot10^{-3}$ l)	20*	$13.333\cdot10^{3}$ €/l
$N_2(sN)_2N_2$ (Sigma-Aldrich, 0.05 µmol, desalted, dry in tubes)	6.74	$0.135\cdot10^{9}$ €/mol (=0.135 €/nmol)
dNTP-Set 1 (Carl Roth, ON: K039.2) (5x 4 dNTPs with 25 µmol each)	419	$1.676\cdot10^{7}$ €/mol
BSA, Molecular Biology Grade (NEB, ON: B9000S) (20 µg/µl, 12 mg=0.012 g)	32	$2.667\cdot10^{3}$ €/g
EquiPhi29™ DNA Polymerase / EP29 DNAP (Thermo Scientific, ON: A39391) (10 U/µl, 1000 U)	254	0.254 €/U
Pyrophosphatase, inorganic (*E. coli*) / iPPase (NEB, ON: M0361S) (0.1 U/µl, 10 U)	66	6.6 €/U
1,4-dithiothreitol / DTT (Carl Roth, ON: 6908.1) (5 g; n=m/M=5 g/154.2 g/mol=0.032 mol)	58.90	1841 €/mol

Tab. 12: Cost calculations for the RCA (V=1.8 µl)
*The costs for the LCR are not included in the cost calculations for the RCA to determine the individual costs of the RCA step.

**Component**	**c_{final} in the RCA**	**$Z = c_{final} \cdot V$ ($V=1.8 \cdot 10^{-6}$ l)**	**P (see Tab. 11)**	**$K = Z \cdot P = c_{final} \cdot V \cdot P$ in €**
LCR*	/	Not included in the calculation	Not included in the calculation	Not included in the calculation
phi29 buffer	1x	/ ($0.18 \cdot 10^{-6}$ l 10x buffer)	$13.333 \cdot 10^{3}$ €/l	0.002
$N_2(sN)_2N_2$	10^{-4} mol/l (=100 µM)	$1.8 \cdot 10^{-10}$ mol	$0.135 \cdot 10^{9}$ €/mol	0.024
dNTP	10^{-3} mol/l each (=1 mM each dNTP)	$1.8 \cdot 10^{-9}$ mol	$1.676 \cdot 10^{7}$ €/mol	0.03
BSA	0.4 g/l (=0.4 µg/µl)	$7.2 \cdot 10^{-7}$ g	$2.667 \cdot 10^{3}$ €/g	0.002
EP29 DNAP	$3.6 \cdot 10^{5}$ U/l (=0.36 U/µl)	0.648 U	0.254 €/U	0.165
iPPase	4000 U/l (=0.004 U/µl)	$7.2 \cdot 10^{-3}$ U	6.6 €/U	0.048
DTT	0.004 mol/l (=4 mM)	$7.2 \cdot 10^{-9}$ mol	1841 €/mol	<0.0001
MilliQ H_2O	/	Not included in the calculation	Not included in the calculation	Not included in the calculation
**Sum**:	/	/	/	**0.271**

Costs of RCA (without LCR): **0.271 € per 1.8 µl RCA** => **K/V=0.271 €/1.8 µl=0.151 €/µl for every 1.8 µl RCA**

Costs of RCA (with LCR): **K(LCR)+K(RCA)=0.048 €+0.271 €=0.319 € per 1.8 µl RCA with LCR**
=> **K/V=0.319 €/1.8 µl=0.177 €/µl for every 1.8 µl RCA with LCR**

3. Costs for the 3.6 µl PEG DNA precipitation

Tab. 13: List of all components required for the PEG DNA precipitation

**Component**	**Price from 12/28/21 in €**	**Price per unit of the individual component (P)**
Polyethylene glycol 8000 / PEG-8000 (Carl Roth, ON: 0263.1) (500 g)	23.50	0.047 €/g
$MgCl_2 \cdot 6\ H_2O$ (Carl Roth, ON: 2189.1) (1 kg; n=m/M=1000 g/203.3 g/mol=4.919 mol)	30.90	6.282 €/mol

Tab. 14: Cost calculations for the PEG DNA precipitation (V=3.6 µl)
*The costs for the LCR and RCA are not included in the cost calculations for the RCA to determine the individual costs of the precipitation step.

**Component**	**c_{final} in the PEG DNA precipitation**	**$Z = c_{final} \cdot V$ ($V=3.6\cdot10^{-6}$ l)**	**P (see Tab. 13)**	**$K = Z\cdot P = c_{final}\cdot V\cdot P$ in €**
RCA*	/	Not included in the calculation	Not included in the calculation	Not included in the calculation
PEG-8000	130 g/l (=13% (w/v))	$4.68\cdot10^{-4}$ g	0.047 €/g	<0.0001
$MgCl_2$	0.01 mol/l (=10 mM)	$3.6\cdot10^{-8}$ mol	6.282 €/mol	<0.0001
MilliQ H_2O	/	Not included in the calculation	Not included in the calculation	Not included in the calculation
**Sum**:	/	/	/	**<0.0001**

<u>**Costs of precipitation (without LCR+RCA)**</u>: **<0.0001 € per 3.6 µl precipitation**

=> No relevance of the precipitation for the total costs

<u>**Costs of precipitation (wit LCR+RCA)**</u>:

K(prec.)+K(RCA)+K(LCR)≈K(RCA)+K(LCR)=0.048 €+0.271 €=0.319 € per 3.6 µl precipitation + RCA + LCR

4. Costs for the 3.6 µl CFPS

Tab. 15: List of all components required for the CFPS

E. coli* cell extract (CE) and cell extract buffer (CEB) were produced with the same composition and consumption of all reagents like in the original publikation by Sun *et al.* (2013)[3]. Therefore, the originally calculated price per volume (P) for cell extract and buffer by Sun *et al.* (2013)[3] can be used as basis for the cost calculations but P_0=0.0105 $/µl for a 10 µl CFPS from the publikation has to be converted in €, recalculated for the price per CE+CEB (25% (v/v) of the 10 µl are DNA and 75% (v/v) of the 10 µl are CE+CEB)** and adapted to the price development to get the extrapolated P_1 for the year 2020:

P_1 = (P_0/CE-CEB fraction) · (exchange rate) · (price development)
= (0.0105 $/µl/0.75) · 0.88 €/$ · 1,087 = 0.01339 €/µl
= 0.0134 €/µl

(exchange rate: 1 $ ~ 0.88 €[1] from 12/28/21; price increase from 2012 to 2020: 8,7%[2] from 12/28/21; CE-CEB fraction of the 10 µl DNA+CE+CEB: 75%=0.75**)

**In Sun *et al.* (2013)[3] the price for the complete 10 µl CFPS (DNA+CE+CEB) is only built up by the price of CE and CEB with a hypothetical DNA price of 0 $. This means that the price of the 10 µl CFPS with 0.11 $ consists of 25% (v/v) DNA (2.5 µl) with 0 $ and 75% (v/v) CE+CEB (7.5 µl) with 0.105 $≈0.11 $. So, the price per volume (P) of CE+CEB is 0.105 $/7.5 µl=0.014 $/µl.

**Component**	**P_0 from 12/2012[3]**	**P_1 (= extrapolated from P_0 for the year 2020)**
mixture of *E. coli* cell extract and cell extract buffer*	0.0092 €/µl*<br>(~0.0105 $/µl)[3]	0.0134 €/µl

Tab. 16: Cost calculations for the CFPS (V=3.6 µl)

*The costs for the LCR, RCA and precipitation are not included in the cost calculations for the RCA to determine the individual costs of the precipitation step.

**Component**	**c_{final} in the CFPS DNA precipitation**	**Z = c_{final} · V**	**P (see Tab. 15)**	**K = Z·P = c_{final}·V·P in €**
Precipitated, resolved DNA*	/	Not included in the calculation	Not included in the calculation	Not included in the calculation
Cell extract + buffer	/	/<br>(1.8 µl mixture of cell extract + buffer)	0.0134 €/µl	0.0241
**Sum**:	/	/	/	**0.024**

<u>Costs of CFPS (without LCR+RCA+prec.)</u>:
0.024 € per 3.6 µl CFPS => **K/V=0.024 €/3.6 µl=0.007 €/µl for every 3.6 µl CFPS**

<u>Costs of CFPS (with LCR+RCA+prec.)</u>:
K(LCR)+K(RCA)+K(prec.)+K(CFPS)=0.048 €+0.271 €+0.024 €=0.343 € per 3.6 µl CFPS with prec., RCA and LCR
K(prec.)<0.0001€
=> **K/V=0.343 €/3.6 µl=0.095 €/µl for every 3.6 µl CFPS with prec., RCA and LCR**

5. Overview over the costs for the HyperXpress workflow

Tab. 17: Overview over all steps of HyperXpress, the reaction volume in a step V(step), the costs per step K(step) and the costs of the whole workflow K_{total}

**Step**	**LCR**	**RCA**	**prec.**	**CFPS**
**V(step) in µl**	0.612	1.8	3.6	3.6
**K(step) in €**	0.048	0.271	<0.0001	0.024
**K_{total} in €**		0.343		

6. Comparison of costs between HyperXpress and the RCA-CFPS system of Dopp *et al.* (2019)[4]

Relevant data for the calculation of the costs per RCA-CFPS system of Dopp *et al.* (2019)[4]:

- Price per mg sfGFP produced through the RCA-CFPS system: $P=61$ \$/mg$=61\cdot10^3$ \$/g[4]
- Final mass concentration β of sfGFP prduced through the RCA-CFPS system: $\beta=0.25$ mg/ml$=0.25$ g/l[4]
- Volume of the final CFPS reaction to produce sfGFP: $V=15$ µl$=15\cdot10^{-6}$ l[4]
- Exchange rate: 1 \$ ~ 0.88 €[1] from 12/28/21

Calculation of the costs K per RCA-CFPS system of Dopp *et al.* (2019)[4]:

$K = P \cdot (\text{exchange rate}) \cdot m \qquad | \ m=\beta\cdot V$

$K = P \cdot (\text{exchange rate}) \cdot \beta \cdot V$

$= (61\cdot10^3 \ \$/g) \cdot (0.88 \ €/\$) \cdot (0.25 \ g/l) \cdot (15\cdot10^{-6} \ l)$

$= 0.201$ €

=> **0.201 € per 15 µl RCA-CFPS system of Dopp *et al.* (2019)[4]**

=> **K/V=0.201 €/15 µl=0.013 €/µl per 15 µl RCA-CFPS system** of Dopp *et al.* (2019)[4]

Tab. 18: Comparison between HyperXpress and the RCA-CFPS system of Dopp *et al.* (2019)[4]
(V_{total}: Final reaction volume, K_{total}: Costs per reaction of the total system)

	**HyperXpress**	**RCA-CFPS system of Dopp *et al.* (2019)[4]**
**V_{total} in µl**	3.6	15
**K_{total} in €**	0.343	0.201
**K_{total}/V_{total} in €/µl**	0.095	0.013

References:

[1]: https://www.boerse-online.de/devisen/devisenrechner/us-dollar-euro

[2]: https://www-genesis.destatis.de/genesis/online?sequenz=tabelleErgebnis&selectionname=61111-0001&startjahr=1991#abreadcrumb

[3]: Sun, Z.Z., Hayes, C.A., Shin, J., Caschera, F., Murray, R.M., Noireaux, V. Protocols for Implementing an Escherichia coli Based TX-TL Cell-Free Expression System for Synthetic Biology. J. Vis. Exp. (79), e50762, doi:10.3791/50762 (2013).

[4]: Dopp JL, Rothstein SM, Mansell TJ, and Reuel NF. Rapid prototyping of proteins: Mail order gene fragments to assayable proteins within 24 hours. Biotechnology and Bioengineering. 2019;116:667–676. https://doi.org/10.1002/bit.26912
